# Supplementary material for: Nutrition, Physical Activity, and Dietary Supplementation to Prevent Bone Mineral Density Loss: A Food Pyramid
Source: Nutrients. 2021 Dec 24;14(1):74. doi: 10.3390/nu14010074 (PMC8746518; doi:10.3390/nu14010074)
Supplement: Supplementary file 1 [file nutrients-14-00074-s001.zip › nutrients-1519822-supplementary/Table S1. BMI bone.pdf]

Author	Type of study	Study period	Methods	Subjects	End point	Results	Conclusion	Strenght of evidence
Silva et al. (2015) [13]	Prevalence study	2 months	Data collected from dexa analysis, including generalities and body measurements	1871 women average age: 59.2 ± 10.5 years	Risk and protective factors of low bone mineral denisty (BMD) (osteopenia ed osteoporosis)	Linear increase in osteopenia and osteoporosis was observed with advancing age ($p < 0.001$). The Body Mass Index (BMI), however, was inversely associated with reduced BMD ($p < 0.001$)	Menopause and age over 50 years old were risk factors for osteopenia and osteoporosis while BMI greater than 25 was a protective factor.	Moderate
Mazocco et al (2017) [14]	Prevalence study	3 months	Data collected from standardized questionnaire, anthropometric parameters and dexa analysis	393 post-menopausal women, mean age was 59.6 ± 8.2 years	Association between BMI and BMD in postmenopausal women	the normal weight women had 1.2 times the prevalence of osteopenia of obese women (PR = 1.2; CI 95% 1.3–1.5). osteoporosis was present twice more in eutrophic women than in obese women (PR = 2; CI 95% 1.4–2.9)	Obese women had lower prevalence of osteopenia and osteoporosis compared with normal weight women	Moderate
Hassan et al. (2020) [15]	Prevalence study	-	Data collected from dexa analysis, including generalities and body measurements	116 female subjects with age range 25-65 years old	Impact of fat mass and its distribution on bone health	Among pre-menopausal women BMD had highly significant positive correlations with BMI The same correlation was found in the post-menopausal group	Bone health positively correlated with BMI, fat mass	Moderate
Oldroyd et al. (2015) [17]	Prevalence study	15 yrs (1991 to 2014)	Data collected from dexa analysis, including generalities and body measurements. The population was divided categories according to BMI	1263 men aged over 50 yrs. Mean age= 64.76±8.47 years	Association between BMI and bone mineral density in a large male population	Positive association between BMD of both the lumbar spine and femoral neck and BMI was dem- onstrated; lumbar spine coefficient 0.01 ($p < 0.05$), femoral neck coefficient 0.01 ($p < 0.05$)	BMD of both the lumbar spine and femoral neck increases with BMI.	Moderate

Tatsumi et al. (2016) [18]	Observational study	17 months	BMD was measured by quantitative ultrasound and anthropometric measurements were collected	749 healthy Japanese women aged 40–74 years (mean 58±8,7)	Relationship between BMI from youth to older age and BMD	Participants underweight both at 20 and at present had a higher Odds Ratio for osteopenia compared with those with BMI ≥18.5 kg/m2 at 20 and at present. Those underweight only at present also had significantly increased OR of developing osteopenia (OR 2.95; 95% CI, 1.67–5.24)	underweight was associated with increased risk for osteopenia,especially in those who were underweight both at 20 and at present.	Moderate
Lim et al. (2016) [19]	Prevalence study	1 year	Data derived from the collection made for the Korean National Health and Nutrition Examination Surveys (KNHANES). body measurments and BMI were assessed. BMD was evaluated by dxa scanner and low skeletal muscle index (SMI)	Participants included 1767 healthy, ≥ 20-year-old, non-obese premenopausal women	Relationship the between underweight, BMD SMI	appendicular lean mass and the SMI were significantly lower in underweight women compared with normal weight women ($p < 0.001$). The Tscores of the lumbar spine and femur were significantly lower in underweight women compared with normal weight women ($p < 0.001$)	Underweight premenopaual women are at a higher risk of low bone mass and low skeletal muscle.	Moderate
Salamat et al. (2013) [16]	Prevalence study	7 months	Data collected from dexa analysis, including generalities and body measurements	230 men (mean age 62,2±8,1 yrs)	Relation between BMI, weight and BMD in an adult men population	Compared to men with BMI ≥ 25, the age-adjusted odds ratio of osteopenia was 2.2 (95% CI 0.85, 5.93) and for osteoporosis was 4.4 (1.51, 12.87) for men with BMI < 25	both BMI and weight are associated with BMD of hip and vertebrae and overweight and obesity decreased the risk for osteoporosis.	Moderate
Lee et al. (2019) [23]	Observational study	-	Data collected from six prospective cohorts, which has been collecting epidemiologic, clinical, and genomic information since 2003. BMD was measured by DXA scanning	3500 subjects, 1433 man, 2031women. Total Median age: 44.3 ±13.7.	Associations of obesity indices with bone health measures for weight-bearing and non–weight-bearing bones	BMI was positively associated with BMD for weight-bearing bones (0.063 ± 0.016 g/cm) per one standard deviation increase in BMI. The association was weaker for non–weight-bearing bones (BMI on BMD: 0.034 ±0.011 g/cm2)	bone health might gain little from obesity and its effect is mainly caused by the weight of the fat mass	Moderate

Hubel et al. (2019) [20]	Meta-analysis	1 yr literature search	94 meta-analyses on 62 samples published	2,319 patients and 1879 controls	Comparing BMD pretreatment, post-treatment, and weight-recovered female patients with AN with controls	fat-free mass (MD: −1.27 kg, 95% CI: −1.79, −0.75, Q = 5.49 × 10−6) and bone mineral density (MD: −0.10 kg, 95% CI: −0.18, −0.03, Q = 0.01) remained significantly altered.	While the majority of traits returned to levels seen in healthy controls after weight restoration, bone mineral density remained significantly altered in AN patients	High
Villareal et al. (2006) [21]	Randomized clinical study	1yr	All 48 participants were randomized in 3 groups:<br><br>-calories restriction(cr):<br><br>energy intake by 16% during the first 3 months and by 20% during the remaining 9 months with diet.<br><br>-exercise group (EX):<br><br>The same caloric deficit was induced with exercise and no diet<br><br>-control group (HL).<br><br>Data collected from dexa analysis, including generalities and body measurements.	48 adults (30 women, 18 man) mean±SD age, 57±3 years; and mean±SD BMI, 27±2 kg/m2	Exercise-induced weight loss is associated with less bone loss than with calories restriction-induced weight loss	Compared with the HL group, the CR group had decreases in BMD at the total hip (−2.2% ± 3.1% vs 1.2% ± 2.1%; *P* = .02) and in the spine BMD (−2.2% ± 3.3%; *P* = .009)	Body weight changes was correlated with BMD changes in the CR (*R*=0.61; *P*=.007) but not in the EX group.	Moderate
Madeira et al. (2014) [22]	Cross-sectional study	-	Data collected from dexa analysis, including generalities and body measurements	50 obese, Age under 50 subjects. 10 males, 40 females. Mean age 34.6 ± 7.0,	Correlations between body composition, BMD and bone	Lean mass was correlated with BMD at the total femur (p = 0.008) and the radius 33% (p = 0.003). multivariate analysis showed that lean mass remained an independent factor	This data suggest that lean mass might be a predictor of bone health in obese individuals with MS.	Moderate

				mean BMI 39.9 ± 6.3	microstructure	influencing bone microstructure		
Fassio et al. (2018) [25]	Narrative review	-	Sources such as MEDLINE/PubMed, CINAHL, EMBASE and Cochrane Library were used	36 relevant papers about obesity and osteoporosis	Review literature in order to explore the relationship between obesity and osteoporosis	-	Obesity is associated with a higher fracture risk in some sites but may be protective at others.	Low
Tanaka et al. (2012) [24]	Cohort study	18 yrs	Data collected from medical interview, physical examination, body composition Mean Follow-up: 6,7 years	1,614 postmenopausal, over 50 yeras old Japanese women	Correlation between BMI and fracture risk	Incidence rates of vertebral fracture in underweight and normal weight women were significantly lower than overweight or obese women by 0.45 (95 % CI: 0.32-0.63) and by 0.61 (95% CI:0.50-0.74).	Overweight/obesity and underweight are both risk factors for fractures at different sites.	Moderate
Nielson et al. (2011) [26]	Cohort study	2 years	Data collected from standardized questionnaire, anthropometric parameters, dexa analysis. physical performance measures and fracture events were registered	5995 over 65 years male participants. mean BMI was 27 kg/m2	Association between BMI and osteoporotic fractures in men	The hazard ratio (HR) for nonspine fracture was 1.04 [95% confidence interval (CI) 0.87–1.25] for overweight, 1.29 (95% CI 1.00–1.67) for obese I, and 1.94 (95% CI 1.25–3.02) for obese II.	If BMD is held constant, it is associated with an increased risk of fracture.	Moderate
Prieto-Alhambra et al. (2012) [27]	Observational study	1year	Data collected from dexa analysis, including generalities and body measurements.	832775 women aged over 50 yrs from a large database called SIDIAP.These were categorized into underweight/ normal (302414 women), overweight	Relationship between BMI and fracture at different skeletal sites in women aged >50 years	Hip fractures were significantly less common in overweight and obese women than in normal/underweight women ($p< 0.001$). Pelvis fracture rates were lower in the overweight group than in the normal/ underweight ($p < 0.001$) one. obese women were at significantly higher risk of proximal humerus fracture than the normal/ underweight group ($p<0.018$).	The association between obesity and fracture in postmenopausal women is site-dependent	Moderate

				(266798), and obese (263563)				
