# Supplementary material for: Nutrition, Physical Activity, and Dietary Supplementation to Prevent Bone Mineral Density Loss: A Food Pyramid
Source: Nutrients. 2021 Dec 24;14(1):74. doi: 10.3390/nu14010074 (PMC8746518; doi:10.3390/nu14010074)
Supplement: Supplementary file 1 [file nutrients-14-00074-s001.zip › nutrients-1519822-supplementary/Table S10a Vitamin B intake.pdf]

Author	Type of study	Study period	Methods	Subjects	End point	Results	Conclusion	Strenght of evidence
Rejnmark et al. (2008) [136]	cohort study	10 years	food records, body composition	1,869 perimenopausal women	association between intake of folate, vitamin B2 and vitamin B12 intakes changes in BMD and fracture risk	positive correlations between daily intake from diet and from diet plus supplements of folate and BMD at the femoral neck ($P < 0.01$).	A high dietary intake of folate, but not vitamin B2 or B12, exerts positive effects on BMD	Moderate
Dai et al. (2013) [137]	cohort study	13 years	food frequency questionnaire and Food Composition Database.	63257 subjects	association between dietary intakes of B vitamins and hip fracture risk among elderly	statistically significant inverse relationship between dietary pyridoxine intake and hip fracture risk was observed among women (p for trend = 0.002) but not among men.	maintaining adequate intake of pyridoxine may prevent osteoporotic fractures among elderly women.	Moderate
