# Supplementary material for: Nutrition, Physical Activity, and Dietary Supplementation to Prevent Bone Mineral Density Loss: A Food Pyramid
Source: Nutrients. 2021 Dec 24;14(1):74. doi: 10.3390/nu14010074 (PMC8746518; doi:10.3390/nu14010074)
Supplement: Supplementary file 1 [file nutrients-14-00074-s001.zip › nutrients-1519822-supplementary/Table S10b. Vitamin B supplementation.pdf]

Author	Type of study	Study period	Supplementation	Subjects	End point	Results	Conclusion	Strenght of evidence
Carmel, et al. (1988) [145]	Case-control study	20 months	multiple injections of 1000 µg of cyanocobalamin	12 patients with cobalamin deficiency and 10 control subjects	The effect of cobalamin on osteoblast-related proteins	Increased OC and skeletal ALP levels with cobalamin therapy in cobalamin-deficient patients, but no effects on control subjects	osteoblast activity may depend on cobalamin	Moderate
Herrmann et al. (2006) [142]	Randomized controlled trial	2 months	0.4, 1 or 5 mg folic acid / day	61 subjects	The effect of a HCY lowering therapy by folic acid (FA) supplementation on biochemical bone markers in healthy subjects.	Increased serum folate and decreased Hcy during supplementation only in the treatment groups	Short-term FA supplementation does not affect biochemical bone markers in non-osteoporotic subjects with a low folate status.	High
Green et al. (2007) [140]	Randomized controlled trial	2 years	1 mg folate + 500 µg vitamin B12 + 10 mg vitamin B6 / day	135 subjects with tHcy > 15 µmol/l (67 placebo group; 68 vitamin group)	The effect of B vitamins in lowering homocysteine and its effect on bone turnover	plasmatic Homocysteine concentrations 5.2 µmol/l lower in the vitamin group than in the placebo group. No significant differences in either serum BSAP or β-CTX between the two groups.	Supplementation with folate and vitamins B-6 and B-12 lowered plasma homocysteine but had no beneficial effect on bone turnover at the end of 2 years	High
Herrmann et al. (2007) [138]	Double-blind, Randomized controlled trial	1 year	A combination of 2.5 mg folate, 0.5 mg vitamin B12 and 25 mg vitamin B6 / day.	47 subjects Placebo (20 F 3 M): Vitamin group (21 F 3 M)	The effect of a homocysteine (HCY)-lowering treatment in osteoporotic individuals.	No change in BMD, TRAP, CTx, OC and PINP; decrease in urinary DPD in the treatment group. Increased lumbar BMD and decreased OC and PINP in the treated hyperhomocysteinemic sub-group.	B-vitamin supplementation had no consistent effects on bone turnover or BMD. However, the situation may be different in patients with hyperhomocysteinemia.	High
Shahab-Ferdows et al. (2012) [144]	Randomized controlled trial	3 months	A single dose of 1 mg hydroxycobalamin i.m., then 500 µg/d oral vitamin B12	132 F (Vitamin B-12 group n = 70, 39.3 ± 10.1 years - Placebo group n = 62: 35.5 ± 10.2 years)	The effect of high-dose vitamin B-12 supplementation on biochemical, hematological, and functional markers of status in a chronically depleted, nonelderly population	Increased serum vitamin B12 and holoTC, and lowered MMA and tHcy after supplementation; no significant effects on BAP, serum folate, ferritin, and hematological variables.	Supplementation did not affect hematology or bone-specific alkaline phosphatase	High
Keser et al. (2013) [143]	Randomized, double-blind, placebo-controlled intervention trial	4 months	800 µg folic acid + 1000 µg vitamin B12 / day	31 F (Vitamin group n = 17, 75.4 ± 8.0 years - Placebo group n = 14, 75.1 ± 8.2 years)	Impact of folic acid and vitamin B12 supplementation on homocysteine levels and, subsequently, on bone turnover markers in older women with mildly to moderately elevated homocysteine levels.	Significantly lower Hcy concentrations and higher concentrations of serum folate and vitamin B12 and RBC folate in the vitamin group compared to the placebo group after supplementation; no significant differences in serum ALP or CTX between groups before or after supplementation.	The use of folic acid and vitamin B12 as a dietary supplement to improve homocysteine levels could be beneficial for older women, but additional research must be conducted in a larger population and for a longer period to determine if there is an impact of supplementation on bone turnover markers or other indicators of bone health.	High
Salari et al. (2014) [146]	Randomized, double-blind,	6 months	1 mg/day folic acid	31 F (Treatment group n = 17, 63.8 ± 8.1 years -	The power of 6-month folic acid supplementation on	Significant changes in the serum level of vitamin B12 (higher), OC (lower) and urinary CTX (lower	The trend of changes of bone biomarkers after 6 months folic acid supplementation shows that homocysteine concentration and/or folic	High

	placebo-controlled clinical trial			Control group n = 14, 64.2 ± 7.3 years)	homocysteine level and bone metabolism.	increase) (but no in Hcy, BALP and urinary PYD) in the treatment group compared with the control group after 6 months.	acid supplementation have impact on the rate of bone metabolism	
Enneman et al. (2015) [140]	Randomized double-blind, placebo-controlled multicenter trial	2 years	A combination of 500 µg B12, 400 µg folic acid and 600 IU vitamin D / day	2276 (1111 with DXA: 563 placebo, 72.8 ± 5.4 years - 548 intervention, 72.4± 5.6 years and 1165 with QUS: 587 placebo, 73.3 ± 7.3 years - 578 intervention, 73.4± 7.4 years)	The effect of Hcy-lowering vitamin B12 and folic acid treatment on bone mineral density (BMD) and calcaneal quantitative ultrasound (QUS) parameters	No statistically significant differences between the intervention and placebo group for FN-, LS-BMD, SOS and BUA. A small, significant beneficial effect of the intervention on BUA among compliant persons > 80 years.	this study showed no overall effect of treatment with vitamin B12 and folic acid on BMD or QUS parameters in elderly, mildly hyperhomocysteinemic persons, but suggests a small beneficial effect on BUA in persons >80 years who were compliant in taking the supplement.	High
Stone et al. (2017) [139]	Randomized, double-blind, placebo-controlled trial	7,3 years	2.5 mg folic acid + 50 mg vitamin B6 + 1 mg vitamin B12 / day	4810 F (Folate/Vitamin B Group: 2402, 62.6 ± 8.7 years; Placebo Group: 2408, 62.5 ± 8.7 years)	If a daily B vitamin intervention including folic acid (2.5 mg/day), vitamin B6 (50 mg/day), and vitamin B12 (1 mg/day) reduces nonspine fracture risk over 7.3 years of treatment and follow-up	No significant effects of supplementation on non-spine fracture risk, nor on change in markers of bone turnover.	no evidence that daily supplementation with B vitamins reduces fracture risk or rates of bone metabolism in middle-aged and older women at high risk of cardiovascular disease	High
Grieger et al. (2009) [153]	Double blind, randomized, placebo-controlled study	6 months	Placebo (P group) containing: starch maize, cellulose, and magnesium stearate.	92 (32 M-60 F)	The effectiveness of a multivitamin (MV) tablet on nutritional status, quantitative heel ultrasound (QUS), mobility, muscle strength and falls	In the MV vs P group: greater increase for serum 25(OH)D, folate and vit B12; greater increase in QUS; trend towards a 63% lower mean number of falls.	MV supplementation raised serum vitamin B12 and folate concentrations and increased serum 25(OH)D, which was accompanied by an apparent positive effect on bone density. We also found a trend towards a reduction in falls and this could contribute to a reduction in fractures.	High
Martin-Bautista et al. (2010) [150]	Longitudinal, controlled, randomized, and double-blinded study	1 year	0.5 l/day of regular semi-skimmed milk with added vitamins A and D (group C)	72 (15 M-57 F): group C (6 M - 27 F, 48 ± 11 years), group E (9 M - 30 F, 50 ± 12 years)	The replacement of regular milk with fortified milk in hyperlipidemic adults for 1 year would improve bone biomarkers.	After 1 year: significant increase in plasma EPA, DHA, vitamin B6, OPG, RANKL, OPG/RANKL, RBC folate, serum folate, vitamin D, and OC in group E. Significant increase in calcium in both groups. No changes in malondialdehyde, PTH or CTX in either group.	Dietary supplementation with the fortified milk drink improved nutritional status and bone formation markers in adult hyperlipidemic patients.	High
Herrmann et al. (2013) [154]	Randomized, double-blind trial	12 months	1200 IU vitamin D3 + 456 mg calcium carbonate daily (group B)	93 (39 M-54 F) - Group A (B, D, Ca) n= 48, 68 years; Group B (D, Ca) n = 45, 70.5 years	The effects of vitamins D3 and B supplementation on bone turnover and metabolism in elderly people.	Significantly decreased bone turnover markers after 1-year vitamin D3 supplementation; no further improvement with additional application of B vitamins. Increased 25(OH)D and lowered PTH in both groups. Decreased tHcy in group A (no change in group B).	One year vitamin D3 supplementation with or without B vitamins decreased the bone turnover significantly. Vitamin D3 lowered parathormone. The additional application of B vitamins did not further improve bone turnover. The marked tHcy lowering by B vitamins may modulate the osteoporotic risk.	High

Groenendijk et al. (2020) [151]	Randomized controlled trial	6 months	Maintaining usual physical activities and dietary habits	163 (80 intervention group, 61.7 ± 6.3 years: 40 M – 40 F / 83 control group, 60.9 ± 6.0 years: 38 M – 45 F)	The effects of a nutrition plus exercise intervention on serum vitamin B-12 and 25-hydroxyvitamin D [25(OH)D], bone turnover markers, and parathyroid hormone (PTH) concentrations in apparently healthy Chinese middle-aged and older adults.	A significant time × group interaction ($P < 0.001$) was found for serum vitamin B-12 and 25(OH)D concentrations and the bone turnover markers	A fortified milk supplement and exercise intervention successfully improved vitamin B-12 and 25(OH)D concentrations as well as the balance of bone turnover markers of Chinese middle-aged and older adults	High
