# Supplementary material for: Nutrition, Physical Activity, and Dietary Supplementation to Prevent Bone Mineral Density Loss: A Food Pyramid
Source: Nutrients. 2021 Dec 24;14(1):74. doi: 10.3390/nu14010074 (PMC8746518; doi:10.3390/nu14010074)
Supplement: Supplementary file 1 [file nutrients-14-00074-s001.zip › nutrients-1519822-supplementary/Table S11a. Vitamin C intake.pdf]

Author	Type of study	Study period	Methods	Subjects	End point	Results	Conclusion	Strenght of evidence
Ratajczak et al. (2020) [158]	Narrative Review	2020	-	-	Vitamin C (ascorbic acid) may affect bone mineral density and deficiency of ascorbic acid leads to the development of osteoporosis	-	Vitamin C has a pleiotropic effect on the human body. Ascorbic acid may have both a direct influence on cells and tissues, as well as an indirect impact by means of antioxidative mechanisms and the regulation of the gene expression.	Low
Sahni et al. (2008) [162]	Cohort study	1948-1998	Associations of total, supplemental, and dietary vitamin C intake with BMD	334 men and 540 women; the mean age of 75 years	Associations of supplemental/dietary vitamin C intake with BMD at varius sites and 4-y BMD change	Higher dietary Vitamin C intake tended to be associated with lower femoral neck-BMD loss	The dietary rather than the supplement component of vitamin C intakes appeared most strongly protective against bone loss.	Moderate

Zhou et al. (2020) [121]	Metanalysis	October 2019	PubMed, EMBASE, and Cochrane Library databases.	384,464 individuals (13 studies)	Estimates for dietary antioxidant vitamin (vitamins A, C, and E) intake and their effect on fracture risk at various sites.	High antioxidant vitamin intake yielded a protective effect on fracture risk.	Fracture risk at all sites is significantly reduced with increased antioxidant vitamin intake,	High
Sun et al. (2018) [159]	Metanalysis	2018	-	7908 controls and 2899 cases of hip fracture	The association of dietary vitamin C intake and the risk of hip fracture.	Dietary vitamin C was statistically correlated with the risk of hip fracture [overall OR = 0.73, 95% CI = 0.55–0.97, I2 = 69.1%]. A linear dose-response association showed that the increase with vitamin C intake of 50 mg/ day statistically reduced by 5% (OR = 0.95, 95% CI 0.91–1.00, P = 0.05) the risk of hip fracture.	Increasing dietary vitamin C intake can decrease the risk of hip fracture.	High
Malmir et al. (2018) [160]	Metanalysis	between 1988 and 2016	-	106 741 individuals aged 20–103 years	The association between vitamin C intake BMD, as well as risk of fractures and osteoporosis	Greater dietary vitamin C intake was positively associated with BMD at femoral neck (pooled r 0·18; 0·06, 0·30) and lumbar spine (pooled r 0·14; 95% CI 0·06, 0·22); however, significant between-study heterogeneity was found at femoral neck: I2=87·6%, Pheterogeneity <0·001. In addition, it was found a non-significant association between dietary vitamin C intake and the risk of hip	Greater dietary vitamin C intake was associated with a 33% lower risk of osteoporosis (overall relative risk =0·67; 95% CI 0·47, 0·94). Greater dietary vitamin C intake was associated with a lower risk of hip fracture and osteoporosis, as well as higher BMD, at femoral neck and lumbar spine.	High

						fracture		
