# Supplementary material for: Nutrition, Physical Activity, and Dietary Supplementation to Prevent Bone Mineral Density Loss: A Food Pyramid
Source: Nutrients. 2021 Dec 24;14(1):74. doi: 10.3390/nu14010074 (PMC8746518; doi:10.3390/nu14010074)
Supplement: Supplementary file 1 [file nutrients-14-00074-s001.zip › nutrients-1519822-supplementary/Table S11b. Vitamin C supplementation.pdf]

Author	Type of study	Study period	Methods	Subjects	End point	Results	Conclusion	Strenght of evidence
Spoelstra-de Man et al. (2018) [161]	Narrative review	-	-	-	Deficiency of Vitamin C can aggravate the severity of illness and hamper recovery.	-	Large randomized controlled trials are necessary to provide more evidence before wide-scale implementation can be recommended.	Low
Rondanelli et al. (2021) [163]	Narrative review	2021	-	2671 subjects	Correlation between BMD, Vitamin C dietary intake, blood levels, and the effectiveness of Vitamin C supplement in humans.	-	BMD values were found to be approximately 3% higher in women who took supplements.	Low
