# Supplementary material for: Nutrition, Physical Activity, and Dietary Supplementation to Prevent Bone Mineral Density Loss: A Food Pyramid
Source: Nutrients. 2021 Dec 24;14(1):74. doi: 10.3390/nu14010074 (PMC8746518; doi:10.3390/nu14010074)
Supplement: Supplementary file 1 [file nutrients-14-00074-s001.zip › nutrients-1519822-supplementary/Table S12a. Calcium intake.pdf]

Author	Type of study	Study period	Methods	Subjects	End point	Results	Conclusion	Strenght of evidence
Balk et al. (2017) [165]	Sistematic Review	2017	13 electronic databases and requested data from domain experts.	78 studies covering 74 countries.	Calcium intake in various countries	-	Only some Northern European nations have shown that they have adequate calcium intake in their population, while the rest of the world is well below the recommended amount of 1000 mg / day	High
Vannucci et al. (2017) [166]	Observational Study	2016	Daily dietary calcium intake assessed by food frequency questionnaire; Total lumbar spine and femoral neck BMD by DXA and by ultrasound techniques. Interviews for history of fragility fractures.	1000 consecutive adult Caucasian subjects (838 Italian women and 162 Italian men)	Effects of dietary calcium intake on BMD and fragility fractures in a sample of an adult Italian outpatient population	10.4% of the subjects had calcium intake >1000 mg/day. No correlation between calcium intake and BMD. Group with intake <400 mg/day: fracture probability ratio 42% vs group with >400 mg/day: 21%.	In an Italian ambulatory population, daily dietary calcium intake is lower than the recommended intake and a higher fracture risk appears to be associated with a reduced calcium intake. Ad age-adeguate daily calcium intake is strongly recommended in ordet to counteract fragility fractures.	Moderate
Wu et al. (2017) [167]	Meta-Analysis	2016	PubMed and EMBASE	17 trials involving 2537 subjects	The efficacy of calcium intake in preventing BMD decrease among postmenopausal women	Two-year calcium intake of 700, 1200, and 2000 mg/day resulted in a maximum efficacy of BMD of 68.0, 81.3, and 89.6%, respectively	Calcium intake can effectively postpone the tendency of BMD decrease in postmenopausal women. An increased calcium dose contributes to the shortening of the onset time. menopausal women can be administered with a rational dose of 1200 mg/day to reduce bone loss	High
Van Den Heuvel et al. (2018) [168]	Sistematic Review	2018	6 meta-analyzes out of a total of 33 randomized controlled trials and 25 prospective studies. Measurements of BMC or BMD and fracture risk	a total of 426,595 subjects	The role of dairy products in the case of bone mineralisation or in the case of fracture risk.	-	intake of 200-250 ml of milk per day (=quantity of calcium between 240 and 300 mg) is associated with a reduction of 5% or more in the risk of fracture.	High
Vannucci et al. (2018) [169]	Sistematic Review	2018	Review of literature focusing on calcium-rich mineral waters ad a source of calcium and their effects on bone metabolism.	-	The bioavailability of the calcium contained in calcium-rich mineral waters and their impact on bone health	-	Calcium rich mineral water is a valuable calorie-free nutritional source of highly bioavailable calcium, and that it can significantly contribute to achieving the daily requirements of this element.	High
