# Supplementary material for: Nutrition, Physical Activity, and Dietary Supplementation to Prevent Bone Mineral Density Loss: A Food Pyramid
Source: Nutrients. 2021 Dec 24;14(1):74. doi: 10.3390/nu14010074 (PMC8746518; doi:10.3390/nu14010074)
Supplement: Supplementary file 1 [file nutrients-14-00074-s001.zip › nutrients-1519822-supplementary/Table S12b. Calcium supplementation.pdf]

Author	Type of study	Study period	Supplementation	Subjects	End point	Results	Conclusion	Strenght of evidence
Silk et al. (2015) [170]	Systematic review and meta-analysis	2015	-	867 participants	The efficacy of calcium supplementation, with or without vitamin D for improving BMD in healthy males	Significant pooled effects size (ES) for comparison between supplementation and control groups were found. The largest effect was found in total body (ES = 0.644; 95% CI = 0.406–0.883; $p < .001$), followed by total hip (ES = 0.483, 95% CI= 0.255–0.711, $p < .001$), femoral neck (ES = 0.402, 95% CI = 0.233–0.570, $p = .000$) and lumbar spine (ES = 0.306, 95% CI = 0.173–0.440,$p < .001$).	When compared with a control intervention, supplementation with calcium, in combination with vitamin D, has a small to moderate effect on bone mineral density at the femoral neck, lumbar spine, total body and total hip in healthy males.	High
Jakeman et al. (2016) [57]	Meta-analysis	2016	-	8 studies on a total number of 30907 adult subjects	The benefits of calcium and vitamin D supplementation on BMD and risk of fracture	-	Calcium + vitamin D therapy brings improvements in BMD and helps in reducing the risk of general fractures	High
Yao et al. (2019) [117]	Meta-analysis	2019	Combined supplementation with vitamin D (daily doses of 400-800 IU, yielding a median difference in 25[OH]D concentration of 9.2 ng/mL) and calcium (daily doses of 1000-1200 mg)	6 RCTs (49282 participants, 5449 fractures, 730 hip fractures)	The benefits of calcium and vitamin D supplementation on risk of fracture	Combined supplementation with vitamin D and calcium found a 6% reduced risk of any fracture (RR, 0.94; 95% CI, 0.89-0.99) and a 16% reduced risk of hip fracture (RR, 0.84; 95% CI, 0.72-0.97).	-	High
