# Supplementary material for: Nutrition, Physical Activity, and Dietary Supplementation to Prevent Bone Mineral Density Loss: A Food Pyramid
Source: Nutrients. 2021 Dec 24;14(1):74. doi: 10.3390/nu14010074 (PMC8746518; doi:10.3390/nu14010074)
Supplement: Supplementary file 1 [file nutrients-14-00074-s001.zip › nutrients-1519822-supplementary/Table S13a. Phosphorus intake.pdf]

Author	Type of study	Study period	Supplement	Subjects	End point	Results	Conclusion	Strenght of evidence
Gutiérrez et al. (2015) [176]	Cohort Study	2015	1000 mg of phosphorus/d using foods known to be free of phosphorus additives for 1 week (low-additive diet), followed by a diet containing identical food items; however, the foods contained phosphorus additives (additive-enhanced diet)	10 Partecipants, age 32±8 years old	The effect of phosphorus additives on markers of bone and mineral metabolism	After 1 week of the low-additive diet, consuming the additive-enhanced diet for 1 week significantly increased circulating fibroblast growth factor 23 (FGF23), osteopontin, and osteocalcin concentrations by 23, 10, and 11%, respectively, and decreased mean sclerostin concentrations (P 0.05).	The enhanced phosphorus content of processed foods can disturb bone and mineral metabolism in humans.	Moderate
