# Supplementary material for: Nutrition, Physical Activity, and Dietary Supplementation to Prevent Bone Mineral Density Loss: A Food Pyramid
Source: Nutrients. 2021 Dec 24;14(1):74. doi: 10.3390/nu14010074 (PMC8746518; doi:10.3390/nu14010074)
Supplement: Supplementary file 1 [file nutrients-14-00074-s001.zip › nutrients-1519822-supplementary/Table S14a. Mangesium intake.pdf]

Author	Type of study	Study period	Methods	Subjects	End point	Results	Conclusion	Strenght of evidence
Gröber et al. (2015) [181]	Narrative Review	2015	-	-	Correlation between low levels of magnesium and chronic and inflammatory disease, such as osteoporosis.	A magnesium deficiency is associated with a variety of diseases	Magnesium supplementation in those patients can be of benefit in most cases.	Low
Workinger et al. (2018) [187]	Narrative Review	2018	MEDLINE database	-	Discuss the causes of magnesium deficiency, absorption, handling and compartmentalization in the body.	-	If serum magnesium is below 0.85 mmol/L and urinary excretion is below 80 mg/day, it is appropriate to consider magnesium related co-morbidities and risk factors for magnesium deficiency when considering whether a state of magnesium deficiency exists.	Low
Razzaque et al. (2018) [189]	Narrative Review	2017-2018	-	-	Sources of magnesium and Magnesium deficiency	A positive association between dietary magnesium intake and BMD.	Vitamin D can positively influence magnesium absorption and support vitamin D metabolism.	Low
Orchard et al. (2014) [190]	Prospective cohort study	1994-1998	- FFQ plus supplements.<br>- Hip fractures by a medical record review;<br>-other fractures identified by self-report.	73,684 post menopausal women (50-79 y – mean 64 y)	Magnesium intake as a risk factor for osteoporotic fractures and altered BMD	Baseline hip BMD was 3% higher (P , 0.001), and whole body BMD was 2% higher (P , 0.001), in women who consumed 422.5 compared with 206.5 mg of Magnesium/day.	Lower magnesium intake is associated with lower BMD of the hip and whole body, but this result does not translate into increased risk of fractures.	Moderate
Erem et al. (2019) [183]	Narrative Review	-	-	-	Correlation between low levels of magnesium and chronic	There is an interaction between vitamin D and magnesium levels, particularly in the elderly and those with	Magnesium deficiency is associated with chronic diseases,	Moderate

					and inflammatory disease, such as osteoporosis.	osteoporosis, which can have a major impact on human health.	including skeletal disorders	
Schwalfenberg et al. (2017) [182]	Systematic Review	-	Terms searched included Magnesium and various conditions purported to be associated with this required mineral including osteoporosis, asthma, diabetes, eclampsia, cardiovascular disease, and cognitive function	-	The important role of Magnesium in a clinically relevant way	Magnesium is required for conversion of vitamin D into its active form which, in turn, supports calcium absorption and metabolism, as well as normal parathyroid hormone function (Vitamin D sufficiency may then increase absorption of calcium and Magnesium by as much as 300%).	Insufficient Magnesium has been linked to a spectrum of clinical afflictions.	High
Jackson et al. (2018) [193]	Retrospective observational study	Data from NHANES 2005-2016	Using data from the NHANES 2005–2016 and the association between ethnicity and magnesium intake	5682 adults aged ≥65 years in the six NHANES cycles with complete information on magnesium intake	The association between ethnicity and magnesium intake in a large, representative sample of U.S. older adults.	Magnesium intake remained lower among African American older adults (13.0 mg/d, 95% CI: 18.8 to 7.2), and higher among those from other ethnic groups (17.2, 95% CI: 3.8 to 30.5) compared with Caucasian older adults. In addition, a higher intake of magnesium was observed among Hispanic older adults (14.0 mg/d, 95% CI: 7.5 to 20.5) relative to Caucasians.	The majority of U.S. older adults do not meet the RDA for magnesium intake, and ethnic differences in magnesium intake exist. After adjustment for covariates, magnesium intake was significantly lower in African Americans compared with Caucasians, and significantly higher in Hispanics and people from other ethnic groups.	Moderate

Dwyer et al. (2003) [185]	Retrospective study	2002	- in-person-24-h recall<br>- a second nonconsecutive 24-h recall via telephone<br>- FFQ	-	An overview of past and present dietary survey methods	45% of Americans and 60% of adults do not take adequate doses of Magnesium	-	Moderate
Fulgoni et al. (2011) [186]	Retrospective study	2011	- in-person 24-h dietary recall<br>- a second 24-h dietary recall via telephone	18063 participants from NHANES 2003–2004 and 2005–2006	Total usual nutrient intakes for 19 micronutrients from all sources as well as the relative contributions of foods	More of the population had total usual intakes below the estimated average requirement) for Magnesium (45%)	Without enrichment and/or fortification and supplementation, many Americans did not achieve the recommended micronutrient intake levels set forth in the Dietary Reference Intake.	Moderate
Mahdavi-Roshan et al. (2015) [190]	Cross-sectional study	2015	- DXA (for classification in osteoporosis, osteopenia and normal mineral density)<br>- anthropometic measures<br>- blood samples	51 post-menopausal women aged between 50 and 80 years	The mineral status between osteopenic and osteoporotic postmenopausal women	The mean dietary intake of Magnesium in post-menopausal women were significantly lower than recommended dietary allowance.<br>.	Magnesium deficiency can affected bone health. Also, magnesium deficiency is associated with the reduction of the levels of PTH and thus the decrease of vitamin D.	Moderate
Veronese et al. (2017) [192]	Observational (prospective) study	Follow-up period of 8 years	- FFQ<br>- self-reported history	3765 participants (1577 men: 2071 women) with a mean age of 60.6 years	Magnesium intake and the onset of fractures in a large cohort of American men and women	Men and women in the highest quintile reported a significantly lower risk for fracture. Women meeting the recommended Magnesium intake were at a 27% decreased risk for future fractures.	Higher dietary Magnesium intake has a protective effect on future osteoporotic fractures, especially in women with a high risk for knee osteoarthritis.	Moderate
