# Supplementary material for: Nutrition, Physical Activity, and Dietary Supplementation to Prevent Bone Mineral Density Loss: A Food Pyramid
Source: Nutrients. 2021 Dec 24;14(1):74. doi: 10.3390/nu14010074 (PMC8746518; doi:10.3390/nu14010074)
Supplement: Supplementary file 1 [file nutrients-14-00074-s001.zip › nutrients-1519822-supplementary/Table S15a. Iron intake.pdf]

Author	Type of study	Study period	Methods	Subjects	End point	Results	Conclusion	Strenght of evidence
Lu et al. (2020) [198]	Cross-sectional study	2019	- timed-endpoint method with the Beckman/Coulter LX20 analyzer<br>-NHANES<br>-Blood samples<br>-Femoral neck and lumbar spine BMD were measured by the Hologic QDR-4500A fan-beam densitometer<br>-24-h dietary recalls	4000 females with an average age of 27.70 years (SD = 11.88 years)	The associations of iron intake, serum iron and serum ferritin with BMD.	Higher serum ferritin was associated with lower femoral neck and lumbar spine BMD (all adjusted P < 0.05); iron intake and serum iron were not associated with femoral neck and lumbar spine BMD.	The elevated serum ferritin level, but not iron intake and serum iron, were associated with lower BMD at femoral neck and lumbar spine.	Moderate
Valenti et al. (2009) [201]	Observational Study	2008	- DXA<br>-statistical analysis	87 consecutive patients with hemochromatosis (HHC)	Prevalence, clinical characteristics and genetic background associated with osteoporosis in Italian patients with hereditary HHC	Lumbar spine T-score was independently associated with total ALP (p = 0.002), hypogonadism/menopause (p = 0.026), and iron overload (p = 0.033 for ferritin and p = 0.017 for iron removed)	Osteoporosis is observed in a quarter of unselected patients with HHC	Moderate
Chon	Cross-	2008 -	- Korean	- 7300 women	Correlation	Serum ferritin levels were	Increased	Moderate

et al. (2014) [202]	sectional, Study	2010	National Health and Nutrition Examination Survey<br>- Anthropometric measurements<br>- Blood samples<br>- Statistical analysis<br>- DXA	(4229 premenopausal and 3071 postmenopausal)	between levels of serum ferritin with BMD in Korean premenopausal and postmenopausal women	only significantly correlated with BMD on the lumbar spine ($\beta=-0.189$, p-value=0.005) in premenopausal women. Additionally, BMD on the lumbar spine had tended to decrease as serum ferritin quartiles increase (P for trend=0.035)	serum ferritin levels were significantly associated with BMD in premenopausal women, particularly on the lumbar spine, but not in postmenopausal women	
Pan et al. (2017) [199]	Observational Study	1998-2012	-Taiwan National Health Insurance Research Database<br>-blood samples<br>- DXA<br>-Statistical analysis	1,000,000 randomly sampled individuals, 35751 individuals with iron deficiency (case group) anemia and 178755 without IDA (control-group)	The role of iron-deficiency anemia (IDA) as a risk factor for osteoporosis	The risk for osteoporosis was significantly higher in the case than the control group (HR = 1.74; 95% CI = 1.61–1.88). The risk for osteoporosis was even higher for patients with IDA who received intravenous iron therapy (adjusted HR = 2.21; 95% CI = 1.85–2.63). In contrast, the risk for osteoporosis was reduced for patients with IDA who received a blood transfusion (adjusted HR = 1.47; 95% CI = 1.20–1.80).	Prior IDA is a significant and independent risk factor for development of osteoporosis.	Moderate
