# Supplementary material for: Nutrition, Physical Activity, and Dietary Supplementation to Prevent Bone Mineral Density Loss: A Food Pyramid
Source: Nutrients. 2021 Dec 24;14(1):74. doi: 10.3390/nu14010074 (PMC8746518; doi:10.3390/nu14010074)
Supplement: Supplementary file 1 [file nutrients-14-00074-s001.zip › nutrients-1519822-supplementary/Table S16a. Copper intake.pdf]

Author	Type of study	Study period	Methods	Subjects	End point	Results	Conclusion	Strength of evidence
Chaudhri et al. (2009) [204]	Observational study	2008	- Blood samples<br>- DXA<br>- quantitative computerized tomography (QCT)	25 early post-menopausal women (1-8 years) with osteopenia	The correlation between the copper concentrations in plasma and BMD	Correlation BMD Lumbar Spine/Cu: n=25 r=0.79 p<0.001; Correlation cortical BMD Lumbar Spine/Cu (measured by the QCT technique): n=23 r=0.85 p<0.001	Copper plays an important role in bone health.	Moderate
Zheng et al. (2014) [205]	Meta-analysis	1995-2013	PubMed database	830 subjects (5 articles)	The relationship between serum Zinc, Copper or Iron and osteoporosis	Patients with osteoporosis had a lower serum level of Copper than healthy controls (SMD=−0.386, 95% CI=[−0.538, −0.234])	The low serum level of Copper was an important risk factor for osteoporosis.	High
Sadeghi et al. (2014) [206]	Observational study	2014	- Blood Samples<br>-DXA	135 women<br><br>Control group t-score > -1 (n = 51)<br><br>Patients group t-score < -1 (n=49)	The association of plasma zinc, copper and toxic elements of lead and cadmium levels with bone mineral density in Iranian women	T-score > −1: Copper (µg/ml) 1.17 ± 0.11<br>T-score ≤ −1: Copper (µg/ml) 1.39 ± 0.13<br>−1.7 < T-score < −1: Copper (µg/ml) 1.24 ± 0.18<br>−2.5 < T-score < −1.7: Copper (µg/ml)	More extensive study with larger ample size might supply definite results about this association for copper and cadmium< −1.7)	Moderate

						1.46 ± 0.17<br>T-score < −2.5:<br>Copper (μg/ml)<br>1.71 ± 0.46		
Qu et al. (2018) [207]	Cross-sectional study	Data from National Health and Nutrition Examination Survey (2011-2014)	-DXA<br>- questionnaires<br>- blood samples	722 subjects with mean age 56 years	The association between serum copper levels, BMD and total fracture.	Individuals in the lowest category (<98.5 mg/dL) of serum copper concentration had 0.049 g/cm2 lower total femur BMD and 0.045 g/cm2 lower femoral neck BMD compared to those in the second concentration category (98.5 - 114 mg/dL). Individuals in the highest category (<134 mg/dL) of serum copper concentration had an approximately 4-fold increase in risk of total fracture compared to those in the second concentration category.	Moderate serum copper levels are critically important for bone health.	Moderate
