# Supplementary material for: Nutrition, Physical Activity, and Dietary Supplementation to Prevent Bone Mineral Density Loss: A Food Pyramid
Source: Nutrients. 2021 Dec 24;14(1):74. doi: 10.3390/nu14010074 (PMC8746518; doi:10.3390/nu14010074)
Supplement: Supplementary file 1 [file nutrients-14-00074-s001.zip › nutrients-1519822-supplementary/Table S16b. Copper supplementation.pdf]

Author	Type of study	Study period	Supplementation	Subjects	End point	Results	Conclusion	Strenght of evidence
Eaton-Evans et al. (2003) [209]	Cross-sectional study	2 years	3 mg copper as amino acid chelate or a placebo to take daily	73 apparently healthy women, aged 45–56 years, recruited from a general practice in north Belfast<br><br>N=24 suppl<br>N=32 placebo	The effects of copper supplementation over 2 years on vertebral trabecular bone mineral density (VTBMD)	There was no difference in initial and final VTBMD for the 24 women who took the copper supplement (initial VTBMD 124.6 (32.1) mg/cm^3 and final VTBMD 123.8 (36.3) mg/cm^3) while the 32 women who took the placebo had significantly lower VTBMD at the end of the study period (initial VTBMD 120.7 (29.2) mg/cm^3 and final VTBMD 113.2 (26.6) mg/cm^3, paired t test P = 0.01).	Although copper supplementation had no effect on the putative biochemical measurements of copper status, it appeared to have reduced the loss of VTBMD in these middle aged women over a 2 year period.	High
Nielsen et al. (2011) [210]	double-blind, placebo-controlled study	2 years	One group (n = 112) was given a daily supplementation with 600 mg Calcium plus placebo and the other with 600 mg Calcium plus 2 mg of Copper and 12 mg of Zinc, for a period of 2 years	224 postmenopausal women, divided into two groups (112+112)	If increased Copper and Zinc intakes would reduce the risk for bone loss.	Women with Calcium plus Copper and Zinc supplementation showed a significant decrease in BMD and T-score while calcium supplementation alone did not show this decrease. Copper intakes <0.9 mg/d were associated with decreased DXA bone status measurements	Long-term low intakes of these elements may increase the risk of osteoporosis	High
