# Supplementary material for: Nutrition, Physical Activity, and Dietary Supplementation to Prevent Bone Mineral Density Loss: A Food Pyramid
Source: Nutrients. 2021 Dec 24;14(1):74. doi: 10.3390/nu14010074 (PMC8746518; doi:10.3390/nu14010074)
Supplement: Supplementary file 1 [file nutrients-14-00074-s001.zip › nutrients-1519822-supplementary/Table S17a. Zinc intake.pdf]

Author	Type of study	Study period	Methods	Subjects	End point	Results	Conclusion	Strenght of evidence
Lowe et al. (2002) [213]	Position paper	/	- 4d weighed diet intake diary.<br>- blood samples<br>- DXA	– 12 elderly postmenopausal women, diagnosed with osteoporosis<br>– Age 62-67y<br>– Ca intake 660-1463 mg/d<br>– BMI 20,4-29,1 kg/m^2	The potential benefits of Copper and Zinc supplementation in reducing bone loss	The use of stable isotopes, coupled with compartmental modelling, has been shown to provide a novel approach to the study of changes in trace mineral metabolism and whole-body status.	Some elderly groups may have reduced dietary intakes and/or absorption of these minerals, and that dietary supplements of Zinc and Copper can effectively reduce the rate of bone loss.	Low
Hyun et al. (2004) [217]	Cohort prospective population-based study	4 years	- Willett Diet Assessment questionnaire<br>- blood samples<br>- DXA	- 396 men (age: 45–92 y)	The independent association between dietary zinc and plasma zinc and the association of each with BMD and 4-y bone loss in community older men.	Dietary zinc intake and plasma zinc concentrations were lower in men with osteoporosis at the hip and spine than in men without osteoporosis at those locations. BMDs for the hip, spine, and distal wrist were significantly lower in men in the lowest plasma zinc quartile (11.3mol/L) than in men with higher plasma zinc concentrations	Dietary zinc intake and plasma zinc each have a positive association with BMD in men.	Moderate
Mutlu et al. (2007) [215]	Cross-sectional study	/	- DXA<br>- Blood samples	- 120 post-menopausal women<br>- Age: 43-80 y<br>- 40 had osteoporosis, 40 had osteopenia and 40 had normal bone mineral density	Changes in Magnesium, Zinc and Copper in groups of postmenopausal, osteoporotic, osteopenic and normal women	Mean concentration of zinc were significantly lower in osteoporotic women than in both osteopenic women and normal women. In addition, zinc concentration in osteopenic women were significantly lower than in normal women.	Trace element supplementation, especially with magnesium and zinc and perhaps copper, may have beneficial effects on BMD	Moderate
Mahdavi-Roshan et al (2015) [190]	Cross-sectional study	/	- DXA (for classification in osteoporosis, osteopenia and normal mineral density)<br>- anthropometic measures<br>- blood samples	- 51 post-menopausal women<br>- Age between 50 and 80 years.<br>- From 51 participants, 23 women had osteoporosis and 28 women had osteopenia.	The mineral status between osteopenic and osteoporotic postmenopausal women in Tabriz, Islamic Republic of Iran.	No statistically significant differences were observed between the osteopenic and osteoporotic groups with respect to serum levels of zinc.	Mineral supplementation especially with calcium, magnesium, zinc and perhaps copper may have beneficial effect on bone density in post-menopausal women with low bone density. Future studies are recommended.	High
Ceylan et al. (2020) [214]	Meta-analysis and Systematic Review	From 1994 to 2020	PubMed, WoS, and Scopus darabases	2899 articles	The effects of zinc supplementation or dietary zinc intake on serum zinc levels and bone turnover markers.	Serum zinc level was significantly lower in osteoporosis subgroup compared with controls (REM p 0.0002, mean difference − 12.68 [− 19.31, − 6.05]. However, there was no any difference between osteoporosis and healthy controls in dietary zinc intake (REM p 0.99, mean difference − 0.01, [− 1.60, 1.57]).	Serum zinc level and dietary zinc intake could have an essential role in preventing osteoporosis.	High
