# Supplementary material for: Nutrition, Physical Activity, and Dietary Supplementation to Prevent Bone Mineral Density Loss: A Food Pyramid
Source: Nutrients. 2021 Dec 24;14(1):74. doi: 10.3390/nu14010074 (PMC8746518; doi:10.3390/nu14010074)
Supplement: Supplementary file 1 [file nutrients-14-00074-s001.zip › nutrients-1519822-supplementary/Table S17b. Zinc supplementation.pdf]

Author	Type of study	Study period	Supplementation	Subjects	End point	Results	Conclusion	Strenght of evidence
Mahdavi Roshan et al. (2013) [216]	Randomized study, double-blind, placebo controlled clinical trial	60 days	- Intervention group (n= 30) receiving 1 capsule of zinc sulfate 220 mg (containing 50 mg elemental zinc) each day.	- 60 postmenopausal osteoporotic women<br>- Age between 48–89 years.	The effect of zinc supplementation on serum zinc and calcium concentrations in postmenopausal osteoporotic women.	In the intervention group serum zinc levels were significantly higher after 60 days [120.5 (SD 7.5) versus 70.5 (SD 4.6) µg/dL] while serum calcium levels were unchanged [8.6 (SD 0.1) versus 9.1 (SD 0.3) mg/dL].	60 days of zinc supplementation had a beneficial effect on zinc levels.	High
Ceylan et al. (2020) [214]	Meta-analysis and Systematic Review	From 1994 to 2020	***Nielsen 2004*** Case-control Zinc supplementation (mg/day): 2 years/3 mg Cu, 53 mg Zn.<br><br>***Nielsen 2011*** Case-control Zinc supplementation (mg/day): 2years/600mg Ca, 2 mgCu, and 12mg Zn<br><br>***Kruger 2009*** Case-control Zinc supplementation (mg/day): 4 months/1200 mg Ca, 96 mg Mg, 2.4 mg Zn, 9.6 µg vitamin D<br><br>***Kruger 2015*** Case-control Zinc supplementation (mg/day): 4months/1200mg Ca, 96mg Mg, 2.4mg Zn, 15µg vitamin D<br><br>***Braam 2003*** Case-control Zinc supplementation (mg/day):3years/500 mg calcium,10 mg zinc, 150 mg magnesium, and 8 mg vitamin D	No restrictions imposed on age, gender, or on any other population characteristic such as race or BMI.	The effects of zinc supplementation or dietary zinc intake on serum zinc levels and bone turnover markers.	Zinc supplementation was effective on the femoral neck and lumbar BMD. Femoral neck BMD was higher in zinc supplementation groups, lumbar BMD was affected negatively. While serum ALP levels were found to be higher with zinc supplementation groups compared with control groups, serum BAP levels did not show the difference between groups , notably serum osteocalcin levels were lower in supplementation groups compared with controls in the random effect model.	Zinc supplementation might improve bone turnover markers for bone formation such as serum osteocalcin and serum alkaline phosphatase, and also BMD, especially on the femoral neck.	High
