# Supplementary material for: Nutrition, Physical Activity, and Dietary Supplementation to Prevent Bone Mineral Density Loss: A Food Pyramid
Source: Nutrients. 2021 Dec 24;14(1):74. doi: 10.3390/nu14010074 (PMC8746518; doi:10.3390/nu14010074)
Supplement: Supplementary file 1 [file nutrients-14-00074-s001.zip › nutrients-1519822-supplementary/Table S18a. Silicon intake.pdf]

Author	Type of study	Study period	Methods	Subjects	End point	Results	Conclusion	Strenght of evidence
Rondanelli et al. (2021) [224]	Narrative review	2021	- FFQ<br>- DXA<br>- measurement of the urinary markers of bone resorption	9644 subjects (men and women)	The effectiveness of Silicon dietary intake in order to suggest a daily dosage of Silicon intake on bone mineral density	An adequate intake in order to promote beneficial effects for bone could be considered to be around 25 mg Silicon/day	Silicon is an essential micronutrient for bone health	Low
