# Supplementary material for: Nutrition, Physical Activity, and Dietary Supplementation to Prevent Bone Mineral Density Loss: A Food Pyramid
Source: Nutrients. 2021 Dec 24;14(1):74. doi: 10.3390/nu14010074 (PMC8746518; doi:10.3390/nu14010074)
Supplement: Supplementary file 1 [file nutrients-14-00074-s001.zip › nutrients-1519822-supplementary/Table S18b. Silicon supplementation.pdf]

Author	Type of study	Study period	Supplementation	Subjects	End point	Results	Conclusion	Strenght of evidence
Rondanelli et al. (2021) [224]	Narrative review	2021	Orthosilicic acid (6 mg), calcium and vitamin D	390 subjects (men and women)	The effectiveness of Silicon dietary supplementation(alone or with other micronutrients), in order to suggest a daily dosage of Si supplementation, on bone mineral density	A combined treatment with orthosilicic acid (6 mg), calcium and vitamin D has a potentially beneficial effect on femoral BMD compared to only use of calcium and vitamin D	Silicon is an essential microelement for bone health	Low
