# Supplementary material for: Nutrition, Physical Activity, and Dietary Supplementation to Prevent Bone Mineral Density Loss: A Food Pyramid
Source: Nutrients. 2021 Dec 24;14(1):74. doi: 10.3390/nu14010074 (PMC8746518; doi:10.3390/nu14010074)
Supplement: Supplementary file 1 [file nutrients-14-00074-s001.zip › nutrients-1519822-supplementary/Table S19a. Manganese intake.pdf]

Author	Type of study	Study period	Supplementation	Subjects	End point	Results	Conclusion	Strenght of evidence
Rivera et al. (2001) [238]	Randomized Controlled Trial	12 months	A supplement contained the recommended dietary allowance for children aged 1. The supplement also contained 1.2 times the RDA for children aged 1–3 y of vitamin A and 1.5 times the RDA of ascorbic acid, riboflavin, vitamin B-12, iron, and zinc.	337 children, average of 12.2 ± 1.8 month	The effect of multiple micronutrient supplementation on the growth of children aged 8-14 months whose diets were poor in several micronutrients	Length gain was almost 5 mm (length-for-age z score: 0.19) greater than the gain in the placebo group. In the younger age group, supplementation resulted in an increase in length of 8.3 mm and in the length-for-age z score of 0.3 units., in the children aged ≥12 mo, the micronutrient group gained only 2.0 mm more in length (a greater length-for-age z score of ≈0.1) than did the placebo group	In the infants, micronutrient supplementation had a positive effect on length gain. Most of the length gain (7.9 mm) occurred during the first 3 periods of supplementation.	High
Friedman e al. (1987) [232]	Cohort study	70 days	- A baseline diet (2.59 mg Manganese/d, 135 mg cholesterol, P:S = 0.86).<br>- After: a formula diet containing 0.11 mg of Manganese/ d for 39 days. Manganese supplements were given to increase the dietary intake to 1.53 and 2.55 mg/d for two 5-days repletion periods following	7 men, 19-22 years	Correlation between calcium, phosphorus and alkaline phosphatase concentrations in serum with manganese depletion.	Calcium before depletion 9,93 mg/dL ±0,55, after depletion 10,8±0,47 mg/dL.<br>-Phosphorus before 3,94±0,29 mg/dL after depletion 4,51±0,25 mg/dL<br>-Alkaline phosphatase before depletion 74,0±24,4 U/L after depletion 100±26,6 U/L	Obbligatory losses may have been lower than normal because of a need to conserve body stores of Manganese during the periods of negative balance.	Moderate

			depletion					
Strause et al (1994) [211]	Randomized Controlled Trial	1994	Randomly assigned to treatment groups: 1. placebo 2. calcium, 3 calcium+placebo trace minerals; 4. placebo calcium, active trace minerals,5. Active calcium, placebo trace minerals; and 6. Active calcium, active trace minerals. Each active supplement contained 15.0 mg of zinc assulfate salt, 2.5 mg of copper, and 5.0 mg of manganese as gluconate salts	59 healthy older postmenopausal women	The correlation between calcium and oligoelements supplementation on bone in postmenopausal women	The overall ANOVA for percent change in spinal bone density was significant (P = 0.033), as was the main effect for calcium (P = 0.045). Loss of bone density was greatest in the placebo group and was significantly different (P = 0.0061) compared with the base-line value .	Loss of bone density was greatest in the placebo group in comparing the four treatment groups, calcium plus trace minerals was the only treatment that differed significantly from placebo (P = 0.0099).	High
