# Supplementary material for: Nutrition, Physical Activity, and Dietary Supplementation to Prevent Bone Mineral Density Loss: A Food Pyramid
Source: Nutrients. 2021 Dec 24;14(1):74. doi: 10.3390/nu14010074 (PMC8746518; doi:10.3390/nu14010074)
Supplement: Supplementary file 1 [file nutrients-14-00074-s001.zip › nutrients-1519822-supplementary/Table S2. Physical activity bone.pdf]

Author	Type of study	Study period	Methods	Subjects	End point	Results	Conclusion	Strenght of evidence
Silva et al. (2015) [13]	Prevalence study	2 months	Data collected from dexa analysis, including generalities and body measurements	1871 women average age: 59.2 ± 10.5 yrs	Risk and protective factors of low BMD (osteopenia ed osteoporosis)	Linear increase in osteopenia and osteoporosis was observed with advancing age ($p < 0.001$). The BMI, however, was inversely associated with reduced BMD ($p < 0.001$)	Menopause and age over 50 years old were risk factors for osteopenia and osteoporosis while BMI greater than 25 was a protective factor.	Moderate
Fuchs et al. (2001) [31]	Randomize d controlled trial	2001	7 months, jumping ex 10 min, 3 x week, progressed from 50 to 100 2-footed jumps from 61 cm high boxes.<br><br>BMC and BMD DXA-derived.	Girls and boys, Asian and white;<br><br>mean age 7.6 +- years,<br><br>randomised;<br><br>Ex: n=45<br><br>C: n=41	A positive correlation between weight-bearing exercise and bone mineral accrual in children and adolescents	LS BMC: +3.1% P<0.05 ** 2.7%<br><br>LS aBMD: +2.0% P<0.01 **0.9%<br><br>FN BMC: +4.5% P<0.001 **3.9%	Results suggest that high impact weight-bearing exercise can benefit bone mineral accrual in this population.	High
Bradney et al. (1998) [32]	Randomize d controlled trial	1998	8 months, 30 min, 3 x week activities included aerobics, foodball, dance, gymnastic, volleyball, basketball, weight training;<br><br>DXA	Boys white, mean age 10.4 years, randomised, bone age, biochemistry and tanner stage to assess maturity;<br><br>Ex: n=19	A positive correlation between weight-bearing exercise and bone mineral accrual in children and adolescents	TB BMD: +1.2%, P<0.01 **0.9%<br><br>LS BMD: +2.8%, P<0.01 **2.1%<br><br>Femoral mid-shaft: +5.6%, P<0.05 **4.2%<br><br>Cortical thickness: +6.4%, P<0.05 **4.8%	Results suggest that moderate impact weight-bearing exercise can benefit bone mineral accrual in this population.	High

				C: n=19				
Kontulainen et al. (2002) [33]	Clinical trial	2002	20-month follow-up after 9-month jumping intervention.<br><br>Both bone mineral content (BMC) misurated by dual energy X-ray absorptiometry (DXA) at the lumbar spine and proximal femur.	99 girls mean age 125<br><br>Trainees n=50<br><br>Controls n=49	20-month follow-up to assess the effect of 9-month jumping intervention on bone gain and physical performance in 99 girls one year after the end of intervention	The trainees (N = 50) had 4.9 % (95 % CI, 0.9 % to 8.8 %, p = 0.017) greater BMC increase in the lumbar spine than the controls (N = 49). The mean 20-month BMC increase in the lumbar spine was 28 % (SD 19) in the trainees compared to 22 % (12) increase in the controls. In the proximal femur, the trend was similar but the obtained 2 to 3 % higher BMC accrual in the trainees (compared to that in controls) were statistically insignificant.	Although the greatest proportion of bone mineral accrual in growing girls is attributable to growth, an additional bone gain achieved by jumping training is maintained at the lumbar spine at least a year after the end of the training.	Moderate
Nilsson e al. (2009) [36]	Randomized controlled trial	2009	Subjects randomly identified using national population registers; standardized questionnaire for collecting information about sport activity (frequency, onset, duration); a p-QCT device was used to scan the distal leg and the distal arm; The cortical volumetric BMD (vBMD; not including the bone marrow; mg/cm3), cortical cross-sectional area (CSA, mm2), endosteal and periosteal circumference (EC and PC, mm), and	1068 men (>18 and <20 years of age).<br><br>Subjects were divided into always inactive and three equal groups (previously sports active), according to the duration of inactivity: group 1, >0 and +-2.24 yr (*n*=95); group 2, >2.24 and +-4.26 yr (*n*=95); group	If physical activity during growth is associated with cortical bone geometry in currently inactive young men	Subjects who continued to be active (*n*=678) and who had been previously active (*n*=285) in sports had a wider cortical bone (periosteal circumference [PC], 4.5% and 3.2%, respectively) with increased cross-sectional area (CSA; 12.5% and 6.9%) of the tibia than the always inactive subjects (*n*=82). Subjects, who ceased their sport activity for up to 6.5 yr previously, still had greater cortical PC and CSA of the tibia than always inactive subjects.	In our study, we showed that currently physically inactive men who had been active in sports during growth had greater cortical cross-sectional area, cortical thickness, and cortical periosteal circumference of the tibia than subjects who had never trained. The results indicate also that sport activity during growth confers positive effects on bone geometry even though sport activity is ceased.	High

			cortical thickness (mm) were measured using a scan	3, >4.26 yr (*n*=95). A > always inactive, B > group 3.				
Baxter-Jones et al. (2008) [35]	Longitudinal study	1991-1997<br>Follow-up 2002-2006	Physical activity questionnaire for children (PAQ-C) and for adolescents (PAQ-A); BMC of the total body (TB), lumbar spine (LS), total hip (TH) and femoral neck (FN) misurated from 1991 to 1997 and 2002 to 2006 by DXA.	154 subjects (82 females and 72 males), entry age 8 to 15 years. Participants returned for follow-up as young adults (follow-up age 23-30 years)	Whether physically active adolescents maintained their higher BMC into the third decade of life when compared to their less active peers.	Active males had 8% greater adjusted BMC at the TB, 13% at the LS and 11% at the TH (p<0.05) in adolescence. Active females had 8% and 15% more adjusted BMC (p<0.05) at the TB and LS. Active adolescents had 8–10% (males) and 9% and 10% (females) more adjusted BMC at the TB, TH and FN (p<0.05).	The results of the study suggest that the promotion of physical activity during adolescence and young adult hood is well advised as a strategy to reduce lifetime risk of osteoporosis and related fractures.	Moderate
Watson et al. (2018) [37]	single-blind, randomized, controlled, exercise intervention trial	From May 2014 to August 2016	Eligible participants were randomized to 8 months of 30-minute, twice-weekly, supervised HiRIT, or unsupervised low-intensity home-based exercise (CON), with an allocation ratio of 1:1. Skeletally non-dominant FN and LS BMD (g/cm2) were obtained using DXA.	101 postmenopausal women older than 58 years with low bone mass (T-score < -1.0 at the hip and/or spine)<br>HIRIT n=49<br>CON n=52	The efficacy and to monitor adverse events of an 8-month, brief, supervised HiRIT program for bone and functional outcomes for postmenopausal women with low to very low bone mass.	HiRIT (n¼49) effects were superior to CON (n¼52) for lumbar spine (LS) BMD (2.9_2.8% versus –1.2_2.8%, p<0.001), femoral neck (FN) BMD (0.3_2.6% versus –1.9_2.6%, p¼0.004), FN cortical thickness (13.6_16.6% versus 6.3_16.6%, p¼0.014)	Brief HiRIT program enhances indices of bone strength and functional performance in postmenopausal women with low bone mass. HiRIT was efficacious this sample of otherwise healthy postmenopausal women with low to very low bone mass.	High
Hind et al. (2007) [34]	Systematic review	2006	Exercise intervention trials, BMD and BMC by DXA as the primary outcome	Randomised and non-randomised controlled	The effects of exercise on bone mineral accrual in	Mean increase in bone parameters over 6 months were 0.9-4.9% in prepubertal, 1.1-5.5% in early pubertal and 0.3-	All trials in early pubertal children, 6 in pre pubertal and 2 in pubertal children reported positive effects of exercise on bone (p<0.05).	High

			measure; secondary outcome measure were structural bone parameters such as hip structural analysis.	trials. Total of 22 trials: 9 in prepuberal children, 8 in early pubertal and 5 in pubertal	children and adolescents	1.9% in pubertal exercise compared to controls ($p<0.05$).		
Troy et al. (2018) [28]	Narrative review	2018	wild range of different workouts, serum and urine bone turnover markers, DXA and computer tomography (CT)	Based on evidence from controlled clinical trials and meta-analyses (randomized/ nonrandomized)	The evidence linking exercise and physical activity to bone health in women	-	Physical activity is an important contributor to bone quality	Low
Cauley et al. (2020) [38]	Systematic review	Studies published between 2009 and 2019	Exercise intervention trials, DXA and computer tomography (CT)	Evidence from 18 prospective observational studies with fracture outcomes and randomised controlled trials of physical activity interventions.	The associations between physical activity and skeletal health in adults older than 40 years.	-	Results suggest an 11-40% decline in fracture risk with increasing physical activity. The effects of exercise on fractures, falls or BMD in older adults could be both site-specific and exercise mode-specific.	High
