# Supplementary material for: Nutrition, Physical Activity, and Dietary Supplementation to Prevent Bone Mineral Density Loss: A Food Pyramid
Source: Nutrients. 2021 Dec 24;14(1):74. doi: 10.3390/nu14010074 (PMC8746518; doi:10.3390/nu14010074)
Supplement: Supplementary file 1 [file nutrients-14-00074-s001.zip › nutrients-1519822-supplementary/Table S20a. Boron intake.pntation.pdf]

Author	Type of study	Study period	Methods	Subjects	End point	Results	Conclusion	Strength of evidence
Boyacioglu et al. (2018) [247]	Cross-sectional study	2014	Boron quantification in 24 h urine samples as a measure of Boron intake through drinking water	53 women in postmenopausal period between 50 and 60 years old	Serum osteocalcin levels; expression of osteocalcin gene rs1800247 polymorphism	Serum osteocalcin levels in postmenopausal subjects in the boron exposed regions: 27.55±3.20 ng/ml.<br><br>Serum osteocalcin levels in control group: 23.47±12.55 ng/ml.	Serum osteocalcin levels in postmenopausal subjects in the boron exposed regions were significantly higher compared to that of control group.<br><br>No statistically significant difference in the osteocalcin genotypes distribution and allele frequency between the groups.	Moderate
