# Supplementary material for: Nutrition, Physical Activity, and Dietary Supplementation to Prevent Bone Mineral Density Loss: A Food Pyramid
Source: Nutrients. 2021 Dec 24;14(1):74. doi: 10.3390/nu14010074 (PMC8746518; doi:10.3390/nu14010074)
Supplement: Supplementary file 1 [file nutrients-14-00074-s001.zip › nutrients-1519822-supplementary/Table S20b. Boron supplementation.pdf]

Author	Type of study	Study period	Supplementation	Subjects	End point	Results	Conclusion	Strength of evidence
Rondanelli et al. (2020) [248]	Narrative review	2020	3 mg/day of boron alone or in combination with other micronutrients	11 clinical trials (594 subjects)	The effectiveness of boron supplementation (alone or with other micronutrients) on growth and maintenance of bone in humans through control of calcium, vitamin D and sex steroid hormone metabolism	A supplementation is demonstrably useful to support bone health, also considering the daily dose of 3 mg is much lower than the Upper Level indicated by EFSA in the daily dose of 10 mg.	A boron supplementation is effective on bone health	Low
