# Supplementary material for: Nutrition, Physical Activity, and Dietary Supplementation to Prevent Bone Mineral Density Loss: A Food Pyramid
Source: Nutrients. 2021 Dec 24;14(1):74. doi: 10.3390/nu14010074 (PMC8746518; doi:10.3390/nu14010074)
Supplement: Supplementary file 1 [file nutrients-14-00074-s001.zip › nutrients-1519822-supplementary/Table S21b. Selenium intake.pdf]

Author	Type of study	Study period	Methods	Subjects	End point	Results	Conclusion	Strenght of evidence
Arikan et al. (2011) [256]	Case-control study	2011	- Atomic absorption spectrophotometry.<br>-DXA	107 postmenopausal women divided into 3 groups: 35 healthy -37 osteopenic -35 osteoporotic	Relationship between Selenium and BMD	Plasma Selenium level was similar in all groups ($p > 0.05$)	Selenium did not directly and correlatively influence BMD	Moderate
Beukhof et al. (2016) [255]	Cohort study	Between 1996 and 2016	- Fluorescence spectroscopy.<br>- DXA	387 healthy elderly men (median age 77 years)	How subtle changes in Selenium status affect BMD.	Selenium is positively associated with total BMD and femoral trochanter BMD, femoral neck and ward's BMD.	Selenium status, within the normal European marginally supplied range, is positively associated with BMD in healthy aging men, independent of thyroid function.	Moderate
Wang et al. (2019) [257]	Cross-sectional study	from October 2013 to December 2015	- Food frequency questionnaire.<br>- Scans radiographic absorptiometry.<br>- Multivariable logistic regression.	6267 subjects (mean age: 52.2 ± 7.4 years; 42% women)	Correlation between dietary selenium intake and prevalence of osteoporosis in the general middle-aged and older population in China.	Compared with the lowest quartile, the energy intake, age, gender and BMI-adjusted odds ratios of osteoporosis were 0.72 (95% confidence interval [CI] 0.55-0.94), 0.72 (95% CI 0.51-1.01) and 0.47 (95% CI 0.31-0.73) for the second, third and fourth quartiles of dietary Selenium intake, respectively (P for trend = 0.001).	In the middle-aged and older humans, participants with lower levels of dietary Selenium intake have a higher prevalence of osteoporosis in a dose-response manner.	Moderate
Zeng et al. (2013) [252]	Narrative review	2013	- X-ray fluorescence spectroscopy. -DXA	1144 postmenopausal women	The properties of the Se in antioxidant protection and in the enhancement of the modulation of cell proliferation, molecular functions relevant to bone health.	Higher selenium levels were associated with higher hip BMD at study entry (β - 0.072, P - 0.004) and lower levels of bone formation (osteocalcin: β - 0.101, P 0.001; procollagen type 1 N-terminal propeptide: β -0.074, P - 0.013) and resorption markers (C-telopeptide of type 1 collagen: β - 0.058, P - 0.050; N-telopeptide of type 1 collagen: β - 0.095, P - 0.002).	Selenium, in super-nutritional doses, induces cell cycle arrest, apoptosis, immune function and the prevention of bone resorption through the inactivation of osteoclasts	Low
