# Supplementary material for: Nutrition, Physical Activity, and Dietary Supplementation to Prevent Bone Mineral Density Loss: A Food Pyramid
Source: Nutrients. 2021 Dec 24;14(1):74. doi: 10.3390/nu14010074 (PMC8746518; doi:10.3390/nu14010074)
Supplement: Supplementary file 1 [file nutrients-14-00074-s001.zip › nutrients-1519822-supplementary/Table S22a. Water intake.pdf]

Author	Type of study	Study period	Methods	Subjects	End point	Results	Conclusion	Strenght of evidence
Dahl et al. (2015) [259]	Prospective study	1994-2000	- Poisson regression models<br>- Survey of trace metals	2110916 person-years in men and 2397217 person-years in women: 5433 men and 13493 women aged 50-85 years suffered a hip fracture during 1994-2000	The association between calcium in drinking water and hip fracture, and whether other water minerals modified this association.	Compared to low calcium in drinking water, a high level was associated with a 15% lower hip fracture risk in men (IRR=0.85, 95% CI: 0.78, 0.91) but no significant difference was found in women (IRR=0.98, 95%CI: 0.93-1.02).	An inverse association between calcium in drinking water and hip fracture risk in men.	Moderate
Wynn et al. (2008) [261]	Comparative study	-	- SPSS statistical software Package.	150 European mineral waters were randomly selected on the Internet: only waters with complete nutritional data for SO4, Cl, Na, K, Magnesium and Calcium were selected.<br>For comparison, forty North American mineral waters were randomly chosen.	Examine which components of mineral waters affect their Calcium content and their alkalizing power, in order to define the optimal profile and composition of a water for bone health	North American waters did not reveal significant results because of their low mineralization. In the European waters, twenty-six out of twenty-eight correlations showed a P value of $\leq$ 0,01.	The best waters would be rich in both bicarbonate and mineral cations including Calcium. This also corresponds to waters low in sulfate. This combination is found in 12% of the 150 studied European waters with Ca >200mg/l and bicarbonate >700mg/l	Moderate
Böhmer et al. (2000) [260]	Systematic review and meta-analysis	1966-1998	First, all publications on calcium absorption from mineral waters were identified and, in a second step, studies comparing calcium absorption from mineral waters with that from dairy products.	-	The correlation between the bioavailability of calcium in calcium-rich mineral waters and osteoporosis	Calcium absorption from mineral waters was significantly higher (p = 0.03) than that from dairy products.	Calcium-rich mineral water is a useful source of calcium for obtaining new, higher daily doses of calcium.	High
