# Supplementary material for: Nutrition, Physical Activity, and Dietary Supplementation to Prevent Bone Mineral Density Loss: A Food Pyramid
Source: Nutrients. 2021 Dec 24;14(1):74. doi: 10.3390/nu14010074 (PMC8746518; doi:10.3390/nu14010074)
Supplement: Supplementary file 1 [file nutrients-14-00074-s001.zip › nutrients-1519822-supplementary/Table S22b. Water supplementation.pdf]

Author	Type of study	Study period	Supplementation	Subjects	End point	Results	Conclusion	Strenght of evidence
Meunier et al. (2005) [262]	Randomized, double-blind, placebo-controlled study	6 months	1 liter of a high calcium mineral water (HCaMW) (596 mg Ca/l)/die	- 152 post-menopausal women with a dietary calcium intake < 700mg/day and mean age: 70.1 ± 4.0 years	The effects of the consumption of a high calcium mineral water (HCaMW) on biochemical indices of bone remodeling in postmenopausal women with low Calcium intake.	After 6 months: a significant 14.1% decrease of serum PTH, osteocalcin (–8.6%), bone alkaline phosphatase (–11.5%), serum (–16.3%) and urine (–13.0%) type-1 collagen C-telopeptide in the HCaMW group compared to the placebo group. In women receiving vitamin D in addition to HCaMW the decrease in bone indices was not found to be greater than in women drinking only the HCaMW.	A daily supplement of 596 mg of Calcium through the consumption of 1 l of HCaMW was able to lower serum PTH and the indices of bone turnover in postmenopausal women with a low Calcium intake.	High
