# Supplementary material for: Nutrition, Physical Activity, and Dietary Supplementation to Prevent Bone Mineral Density Loss: A Food Pyramid
Source: Nutrients. 2021 Dec 24;14(1):74. doi: 10.3390/nu14010074 (PMC8746518; doi:10.3390/nu14010074)
Supplement: Supplementary file 1 [file nutrients-14-00074-s001.zip › nutrients-1519822-supplementary/Table S23a. Caffeine intake.pdf]

Author	Type of study	Study period	Methods	Subjects	End point	Results	Conclusion	Strenght of evidence
Massey et al. (1994) [265]	Clinical study	2 weeks	Multiple blood and 24-hour urinary outputs	25 women, (mean age 65 years) consuming at least 200 mg of caffeine daily.	The effect of 2 weeks of caffeine abstinence on calcium and bone metabolism	Subjects with low calcium intake had significantly increased fasting serum ultrafiltrable calcium levels and significantly decreased serum bone alkaline phosphatase levels after abstinence (P value = 0,01)	Abstinence from moderate caffeine intake raises ultrafiltrable Calcium and decreases bone alkaline phosphatase in older women.	Moderate
Lee et al. (2014) [268]	Meta-analysis	-	Data collected from studies found in literature.	253,514 participants with 12,939 fracture cases from 9 cohort and 6 case–control studies	A better quantification of the association between coffee consumption and risk of fractures	The estimated RR of fractures at the highest level of coffee consumption was 1.14 (95% CI: 1.05–1.24; I2 = 0.0%) in women and 0.76 (95% CI: 0.62–0.94; I2 = 7.3%) in men.	Coffee consumption was associated with an increased risk of fractures in women in a dose- dependent fashion.	High
Hallstrom et al. (2006) [266]	Cohort study	Medium follow-up=10,3 years	- Standardized questionnaire<br>- anthropometric parameter<br>- fracture events	31,527 Swedish women aged 40-76 years	An association between high coffee intake and fracture risk in women	A high coffee consumption significantly increased the risk of fracture (p for trend 0.002), this effect was confined to women with a low calcium intake (<700 mg/day	A daily intake of 330 mg of caffeine, or more may be associated with a modestly increased risk of osteoporotic fractures, especially in women with a low intake of calcium	Moderate
Rapuri et al. (2001) [267]	Observational study	3 years	- DXA<br>- blood samples,<br>- body measurements.	489 elderly women (aged 65–77 y)	The status of BMD of postmenopausal women consuming high or low amounts of	In all skeletal sites considered, women with caffeine intakes of >300 mg/d lost more bone than did	A caffeine intake of >300 mg/d increased the rate of bone loss.	Moderate

					caffeine	those with intakes ≤300 mg/d.		
Wikoff et al. (2017) [269]	Systematic review	-	Systematic review of literature on potential adverse effects of caffeine published from 2001 to June 2015	>5000 articles	Potentials adverse effects of caffeine on human health	-	A consumption of up to 400 mg of caffeine/day in healthy adults wass not associated with any kind of alteration.	High
Doepker et al. (2018) [270]	Systematic review	-	Sreview of literature (studies published between June 2001 and June 2015)	5000 articles, 380 for final review evaluation	If caffeine doses not to be associated with adverse effects	-	A lack of effects of caffeine on bone at levels below 400 mg/day, with a moderate level of confidence.	High
