# Supplementary material for: Nutrition, Physical Activity, and Dietary Supplementation to Prevent Bone Mineral Density Loss: A Food Pyramid
Source: Nutrients. 2021 Dec 24;14(1):74. doi: 10.3390/nu14010074 (PMC8746518; doi:10.3390/nu14010074)
Supplement: Supplementary file 1 [file nutrients-14-00074-s001.zip › nutrients-1519822-supplementary/Table S23b. Caffeine supplementation.pdf]

Author	Type of study	Study period	Supplementation	Subjects	End point	Results	Conclusion	Strenght of evidence
Kynast-Gales et al. (1994) [264]	Observational study	2 days	2 caffeine doses of 3 mg/kg lean body mass caffeine	17subjects: 10 women and 7 men.	If later renal conservation occurs in calcium and magnesium excretion after morning caffeine assumption	Caffeine increased net 24-hour Calcium and Na excretion 0.32 mmol ($p < 0.05$) and 22.4 mmol ($p < 0.05$), respectively, while Magnesium excretion increased 0.16 mmol ($p = 0.11$).<br>No significant differences between male and female	Caffeine-induced short-term increase in urinary Ca excretion	Moderate
