# Supplementary material for: Nutrition, Physical Activity, and Dietary Supplementation to Prevent Bone Mineral Density Loss: A Food Pyramid
Source: Nutrients. 2021 Dec 24;14(1):74. doi: 10.3390/nu14010074 (PMC8746518; doi:10.3390/nu14010074)
Supplement: Supplementary file 1 [file nutrients-14-00074-s001.zip › nutrients-1519822-supplementary/Table S24. Salt intake.pdf]

Author	Type of study	Study period	Methods	Subjects	End point	Results	Conclusion	Strength of evidence
Bedford et al. (2011) [275]	Cross-sectional study	5 months	- Telephone interview.<br>- Urine collection<br>- Physical measurements,<br>- FFQ,<br>- statistical analyses	102 non-obese healthy young women (19-35 years old),	The relationship between 24-h urinary sodium and urinary calcium excretion and the association in those with higher *versus* lower calcium intakes.	For every 100 mmol (2300 mg) increase in sodium excretion, calcium excretion increased by 1.1 mmol (44 mg).	Urinary sodium was inversely associated with hip BMD for all participants (r-0.21, p=0.04) and among women with lower but non higher calcium intakes. Assessed a potential relationship between 24-h urinary sodium, calcium, BMD and 2-year BMD change.	Moderate
Kim et al. (2015) [276]	Cross-sectional study	KNHANES 2008, 2009, 2010 and 2011 data.	- biochemical and clinical variables<br>- DXA<br>- standardized health questionnaires<br>- statistical analyses	2779 postmenopausal women 62.7 +-8.8 years old: 1235 osteoporosis (no osteoporosis n=1544)	Urinary sodium excretion association with BMD and prevalence of osteoporosis in postmenopausal women.	High urinary sodium excretion was significantly associated with low BMD and high prevalence of osteoporosis in lumbar spine	High sodium chloride intake decreases BMD and increase the risk of osteoporosis in postmenopausal women.	Moderate
Kim et al. (2017) [277]	Cross-sectional study	Data from 2008-2011 Korea National Health and Nutrition Examination Survey	- biochemical and clinical variables<br>- DXA<br>- standardized health questionnaires<br>- statistical analyses standardized health questionnaires. Statistical analyses	3635 postmenopausal women.<br>1542 participants (42.4%) with osteoporosis	The association between dietary sodium intake and osteoporosis in Korean postmenopausal women	The prevalence rates of osteoporosis at the lumbar spine were significantly higher in participants who consumed ≥4001 mg of salt than those who consumed ≤2000 mg. At the femoral neck, rates were significantly higher for those who consumed ≥5001 mg	An excessive daily sodium intake is associated with a higher osteoporosis prevalence in Korean postmenopausal women.	Moderate

						compared with those who consumed ≤4000 mg.		
Kwon et al. (2017) [278]	Cross-sectional study	2008 - 2011	Data from the Korea National Health and Nutrition Examination Survey (KNHANES) 2008-2011. Multiple regression analysis. Face-to-face interview, self-administration of health interview questionnaire. Dietary intake data collected by 1-day 24-h recall method during household interview.	9526 women older than 18 years, divided into a premenopausal (n=4793) and postmenopausal (n=4733) group..	The relationship between high intake of sodium and lower bone mass and risk of osteoporosis in adult women.	Postmenopausal women, whose sodium intake was ≥2000 mg/day (odds ratio 1.284, 95% CI 1.029–1.603, P = 0.027), were at risk of developing osteoporosis.	A high sodium intake was negatively associated with BMC/BMD in post-menopausal women, but not in premenopausal women.	Moderate
Fatahi et al. (2018) [273]	Systematic Review	2017	PubMed/medline, SCOPUS and web of science	Cross-sectional and cohort designs. 39,065 subjects	The relation of dietary/urinary sodium with BMD, BMC, and the risk of osteoporosis.	Higher sodium consumption significantly increased the risk of osteoporosis (OR=1.20; 95% confidence interval [CI], 1.02–1.41; p=0.026). higher risk of osteoporosis is in premenopausal women and subjects older than 50 years.	A positive association between sodium intake and the risk of osteoporosis, while there is no association with urinary sodium.	High
