# Supplementary material for: Nutrition, Physical Activity, and Dietary Supplementation to Prevent Bone Mineral Density Loss: A Food Pyramid
Source: Nutrients. 2021 Dec 24;14(1):74. doi: 10.3390/nu14010074 (PMC8746518; doi:10.3390/nu14010074)
Supplement: Supplementary file 1 [file nutrients-14-00074-s001.zip › nutrients-1519822-supplementary/Table S25. Isoflavones supplementation.pdf]

Author	Type of study	Study period	Supplementation	Subjects	End point	Results	Conclusion	Strenght of evidence
Harahap et al. (2021) [46]	Narrative review	2021	136.6 mg aglycone equivalence or 2 soy isoflavone doses (80 or 120 mg/day) or soy isoflavones (with or without calcium) or isoflavone capsules and kiwifruit	360 postmenopausal women, with or without bone diseases	Effect on calcium status and bone health in postmenopausal women with or without bone diseases	Isoflavones attenuate BMD loss, improves bone turnover, interact with serum calcium in affecting whole-body BMD and reduce osteocalcin level	Isoflavones, including their metabolites, increase bone mineral density by stimulating bone formation	Low
Lambert et al. (2017) [287]	Randomized controlled trial	12 months	Red clover extract (RCE) (60 mg isoflavone aglycones/days and probiotics) + calcium (1200 mg/day), magnesium (550 mg/day), and calcitriol (25 µg/day)	78 postmenopausal osteopenic women with 61,8 ± 1,0 years	The beneficial effects of a bioavailable isoflavone and probiotic treatment against postmenopausal osteopenia	RCE significantly attenuated bone mineral density (BMD) loss at the L2-L4 lumbar spine vertebra ($P < 0.05$), femoral neck ($P < 0.01$), and trochanter ($P < 0.01$) compared with placebo	Twice daily RCE intake over 1 y potently attenuates BMD loss caused by estrogen deficiency and improves bone turnover	High
Shedd-Wise et al. (2011) [288]	Randomized, double blind, placebo controlled trial	3 years	Two soy isoflavone doses (80 or 120 mg/day)	171 healthy postmenopausal women (46–63 years)	The effects of soy isoflavone on bone strength in postmenopausal women	As time since last menstrual period (TLMP) increased ($p=0.012$), 120 mg/d was protective of cortical BMD. 80 mg/day became protective as bone turnover increases ($p=0.011$).	Soy isoflavone treatment for 3 years is modestly beneficial for midshaft femur BMD as TLMP increases	High
Zhang et al. (2020) [289]	Randomized, double blind, placebo controlled trial	6 months	Soy isoflavone or calcium and soy isoflavone combined with calcium therapy	160 perimenopausal women with osteoporosis or osteopenia	The effect of placebo, soy isoflavone, calcium and soy isoflavone combined with calcium on BMD	Mean changes from baseline values of BMD, calcium/phosphorus,vitamin D and glutathione peroxidase (GSH-pX) activity are significantly increased	Soy isoflavone, calcium and isoflavone combined with calcium therapy are effective and safe on attenuating BMD loss in perimenopausal women	High
Marini et al. (2007) [290]	Randomized, double blind, placebo controlled	4 weeks + 24 months	54 mg of genistein given daily	389 postmenopausal women with a BMD less than 0.795 g/cm2 at	The effects of genistein on bone metabolism in osteopenic postmenopausal women	BMD increases in genistein recipients at the anteroposterior lumbar spine (change, 0.049 g/cm2 [95% CI, 0.035 to 0.059]. Genistein	24 months of treatment with genistein has positive effects on BMD in osteopenic postmenopausal women	High

	trial			the femoral neck		statistically significantly decreases urinary excretion of pyridinoline and deoxypyridinoline and increases levels of bone-specific alkaline phosphatase		
