# Supplementary material for: Nutrition, Physical Activity, and Dietary Supplementation to Prevent Bone Mineral Density Loss: A Food Pyramid
Source: Nutrients. 2021 Dec 24;14(1):74. doi: 10.3390/nu14010074 (PMC8746518; doi:10.3390/nu14010074)
Supplement: Supplementary file 1 [file nutrients-14-00074-s001.zip › nutrients-1519822-supplementary/Table S3a. CHO intake.pdf]

Author	Type of study	Study period	Methods	Subjects	End point	Results	Conclusion	Strength of evidence
Matsuzaki et al. (2019) [41]	Randomized controlled trial	1 year	QUS	UHHPBR-intake group (n= 20): 100 g of UHHPBR + 100 g of white rice/day; white rice group (n=20): 200 g of white rice/day. Mean age 73,1 years	Long-term intake of UHHPBR prevents aging-related decline of BMD	The calcaneus BAR value in the UHHPBR-intake group was significantly higher than that in the WR-intake group at month 12 (p, 0.05)	Long-term oral intake of UHHPBR prevents BMD decline in the elderly	High
Dai et al. (2018) [42]	Longitudinal study	8 years	food frequency questionnaire (FFQ), DXA	1857 (1,065 women, 792 men)	Association between dietary fiber and bone loss at the femoral neck, trochanter and lumbar spine	In men higher fiber intake was associated with less bone loss at the hip.	Dietary total fiber and fiber from fruits may protect against bone loss at the hip in men but not in women.	Moderate
Lee et al. (2019) [43]	Cross-sectional study	2011	24 h recall	2187 (1306 women, 881 men)	Correlations between daily fiber intake and BMD	In males aged between 18 and 45, fiber intake significantly increased BMDs of L1 (p, 0.040) and L2 (p, 0.038)	Fiber intake is a protective factor of lumbar spine (L1 and L2) BMD in managed 18- 45	Moderate
Zhou et al. (2021) [44]	Prospective cohort study	8.1 years	heel ultrasound, FFQ	384 134 partecipants	Association between dietary fiber intake and BMD	Men showed higher heel-BMD than women (0.521 vs 0.576 g/cm2, P< .001). Dietary fiber score was higher in women compared with men (14.5 vs 14.0 g/d, P < .001).	Higher dietary fiber consumption was as-sociated with higher BMD; the association was more marked in men	Moderate
Villa et al. (2017) [45]	Narrative Review	2017	Literature revision regarding correlation between gut microbiota and bone axis	//	The effect of the gut-associated microbial community on bone health	Gut microbes positively impact bone mineral density and strength parameters	Effectiveness of prebiotics and probiotics is dependent on stage of development	Low

Chen et al. (2006) [47]	Cross-sectional study	Oct 1999-Jan 2001	FFQ, DXA	670 postmenopausal women	Association between fruits and vegetables intake with BMD	Fruit intake was associated with increased BMD of the whole body (P =0·032), spine (P<0·001) and total hip (P=0·074); vegetable intake was positively correlated with the whole body (P= 0·082) and total hip BMD (P=0·025)	Greater fruit and vegetable intake is independently associated with better BMD	Moderate
Li et al. (2014) [48]	Cross-sectional study	Jul 2009-May 2010	FFQ, DXA	222 early adoles-cent boys and girls, 371 parturient women ,333 postmenopausal women	Association of fruit and vegetable consumption with bone health	BMD Z-score increased by 0,25 (or 2,1 % of the mean), 0,22 (3,5 %), 0,23 (3,0 %) and 0,25 (3,5 %), and BMC Z-score increased by 0,33 (5,7 %), 0,25 (5,8 %), 0,34 (5,9 %) and 0,29 (4,7 %), at the total body, lumbar spine, total hip and femoral neck in participants belonging to the top tertile compared with the bottom tertile of fruit intake (all P<0,05) respectively	Fruits and vegetables may have a bone sparing effect	Moderate
Qiu et al. (2017) [49]	Cross-sectional study	2011-2013	FFQ, DXA	3089 middle-aged and elderly Chinese subjects	Consumption of fruit and vegetables may benefit bone health	Mean BMD was higher in tertile 3 vs. tertile 1 by 1.33% (total hip) and 1.31% (femur neck) for FV, and 1.10% (whole body), 1.57% (total hip,And 2.05% (femur neck) for fruit	Greater intake of FV was independently associated with a higher BMD and a lower presence of osteoporosis in middle-aged and elderly Chinese subjects with lower BMI	Moderate
Hoomshmand et al. (2011) [50]	Randomized double-blind cross-over study	1 year	DXA, blood samples	160 postmenopausal women in 2 treatment groups: dried plum (100 g/d) or dried apple (75 g/d comparative control).	Dried plum reverse bone loss in osteopenic postmenopausal women.	Dried plum had more pronounced effects on BMD of ulna and spine; serum BALP levels were significantly reduct at 12 months in dried plum group compared with baseline; serum OC levels were significantly lower in the dried plum group compared with the dried apple group at	Longer-term consumption of dried plums increases BMD in postmenopausal women	High

						3- and 12-month time points; TRAP5b decreased significantly in the dried plum group at 3 months; serum CRP levels were significantly lower in the dried plum group compared with the dried apple group at 3 months		
Hoomshmand et al. (2016) [51]	Randomized double-blind cross-over study	6 months	DXA, blood samples	48 osteopenic women in 3 treatment groups: (1) 50 g of dried plum; (2) 100 g of dried plum; (3) control	Possible dose-dependent effects of dried plum in preventing bone loss in	Both doses of dried plum prevent the loss of total body BMD compared with control group (P<0.05); TRAP-5b decreased at 3 months and this was sustained at 6 months in both 50 and 100 g dried plum groups (P<0.01 and P <0.04, respectively); BAP/TRAP-5b ratio was significantly (P< 0.05) greater at 6 months in both dried plum groups	Lower dose of dried plum (i.e., 50 g) may be as effective as 100 g of dried plum in preventing bone loss	High
Dreher et al. (2018) [52]	Narrative review	2018	Literature revision regarding correlation between whole fruits and human lifecycle	18393 man and women all ages	Health benefits associated with the adequate intake of whole fruits	-	Adequate intake of whole fruit contributing to higher bone mineral density in children and adults	Low
Whisner et al. (2018) [58]	Narrative review	2018	Literature revision regarding prebiotic effects and bone health	1288 man and women all ages	Discuss research in the area of prebiotics, mineral metabolism, and bone health	-	Prebiotic type differentially influences the absorption of different minerals and bone mineral	Low
Sun et al. (2017) [59]	Cross-sectional study	march - december, 2012	QUS, blood samples	4080 postmenopausal Chinese women.	Relationships between osteopenia and dyslipidemia, gly-cemic levels or blood pressure	The prevalence of osteopenia was significantly lower in subjects with FBG 7.0–7.9 mmol/L (0.659 [0.473, 0.918]); FBG ≥ 8.0 mmol/L (0.720 [0.541,	Bone change in diabetic women may be more complicated and need alternative marker to predict the osteopenia.	Moderate

						0.958]); PBG ≥ 15.0 mmol/L (0.690 [0.513, 0.926]); HbA1C 6.5–7.5 % (0.638 [0.495, 0.824]); HbA1C ≥7.5 % (0.715 [0.533, 0.960]).		
Compston et al. (2018) [61]	Narrative review	2018/	Literature revision regarding bone fragility and fractures	-	Epidemiology, pathogenesis and pathophysiology of increased bone fragility and fractures and possible preventive strategies.	-	T2DM is associated with increased risk of fracture, particularly at the hip and BMD underestimates fracture risk in these individuals	Low
