# Supplementary material for: Nutrition, Physical Activity, and Dietary Supplementation to Prevent Bone Mineral Density Loss: A Food Pyramid
Source: Nutrients. 2021 Dec 24;14(1):74. doi: 10.3390/nu14010074 (PMC8746518; doi:10.3390/nu14010074)
Supplement: Supplementary file 1 [file nutrients-14-00074-s001.zip › nutrients-1519822-supplementary/Table S3b. CHO supplementation.pdf]

Author	Type of study	Study period	Supplementation	Subjects (age, sex, number…)	End point	Results (numbers)	Conclusion (descriptive)	Strength of evidence
Harahap et al. (2021) [46]	Narrative review	6-12 months	Multispecies probiotic supplement or three Lactobacillus strains or 10^{10} colony-forming units of L. reuteri or probiotic B. subtilis C-3102	126 women with postmenopause and bone disease	Effects of probiotics on calcium status and bone health	L. reuteri 6475 for 12 months reduced loss of tibia total BMD in older women. C-3102 improves BMD by inhibiting bone resorption and modulating gut microbiota	The probiotics that positively affect bone health are Lactobacillus and Bifidobacterium	Low
Van den Heuvel et al. (1999) [53]	Randomized double-blind cross-over study	three 9 days and two 19-day wash-out periods.	5 g/d and 10 g/d of lactulose	12 postmenopausal women	Effect of lactulose on calcium absorption.	Significant difference in calcium absorption between the highest dose of lactulose and the reference treatment ($p< 0.01$)	Consumption of lactulose increases calcium absorption in a dose-response way.	high
Van den Heuvel et al. (2000) [54]	Randomized double-blind cross-over study	9 days and 19-day wash-out period.	20 g/d TOS	12 postmenopausal women	TOS stimulates Ca absorption	TOS increased true calcium absorption 16%, between reference treatment and TOS treatment	An increased Ca absorption was observed after consumption of a product rich in TOS compared with the reference treatment.	high
Kim et al. (2004) [55]	Randomized double-blind parallel study	3 months	8 g chicory fructan fiber	26 postmenopausal women	Effects of chicory fructan fiber on BMD, absorption of minerals and serum parameters on bone tournover	Calcium absorption increased by 42% in fructan group and decresed by 29% in control group	Intake of chicory fructan fiber in a regular diet increases calcium absorption	high
Whisner et al.	Randomized double-blind	Two 3-week	12 g/d SCF in a low-Ca diet (600	24	Effect of SCF on Ca	Fractional Ca absorption was 12% higher (41	Moderate daily intake of SCF increases Ca absorption	high

(2014) [56]	cross-over study		mg/d).	adolescents	absorption	mg/d) after the SCF treatment	in adolescents with a low Ca intake	
Jakeman et al. (2016) [57]	Randomized double-blind cross-over study	50 d	10 g/d and 20/d SCF	14 postmenopausal women	Skeletal benefits of SCF	In 10g and 20 g fiber from SCF/d, bone calcium retention was improved by 4.8% (P , 0.05) and 7% (P , 0.04)	Chronic doses of 10 and 20 g fiber from SCF/d increased bone-calcium retention dose dependently.	high
