# Supplementary material for: Nutrition, Physical Activity, and Dietary Supplementation to Prevent Bone Mineral Density Loss: A Food Pyramid
Source: Nutrients. 2021 Dec 24;14(1):74. doi: 10.3390/nu14010074 (PMC8746518; doi:10.3390/nu14010074)
Supplement: Supplementary file 1 [file nutrients-14-00074-s001.zip › nutrients-1519822-supplementary/Table S4a. Lipids intake.pdf]

Author	Type of study	Study period	Methods	Subjects	End point	Results	Conclusion	Strength of evidence
Zalloua et al. (2007) [62]	Cross-sectional study	Oct 2013-Jan 2014	FFQ lifestyle habits, DXA	12,055 (5848 men + 6207 women, age 25–64) rural Chinese population	Effects of dietary habits on BMD	Consuming > 250 g /week of seafood is significantly associated with greater BMD in women (p<0.001)	Adequate consumption of fish prevents bone loss and the onset of osteoporosis	Moderate
Kuroda et al. (2017) [63]	Cross-sectional study	-	Diet history questionnaire (DHQ), DXA	275 healthy women (age 19–25	n-3 or n-6 fatty acids play an important role in PBM	Intake of n-3 fatty acids is significantly related to total hip BMD	Adequate total n-3 fatty acid intake may help maximize PBM at the hip	Moderate
Lavado-Garcia et al. (2018) [64]	Cross-sectional study	Feb 2010-May 2013	FFQ, DXA	1865 women (age 20–79)	Relationships between dietary intakes of LCO3-PUFAs and BMD	Dietary intake of ALA is significantly associated with BMD at the hip in osteopenic women, dietary intake of DHA was also significantly associated with BMD at the lumbar spine.	Intake of LCO3-PUFAs seems to be positively associated with BMD at hips and lumbar spine in normal and osteopenic women	Moderate
Maggio et al. (2009) [65]	Narrative Review	2009	Literature revision regarding correlation between omega-3/omega-6 fatty acids ratio and bone health	17721	Higher dietary omega-3/omega-6 fatty acids ratio is associated with beneficial effects on bone health.	-	No definitive conclusions on the therapeutic value of omega 3 fatty acids	Low
Kelly et al. (2013) [66]	Narrative Review	2013	Literature revision regarding LGCI and osteoporosis	-	Relationship between LGCI, fat, bone, and n-3 PUFA.	-	n-3 PUFA positively influence osteoporosis by reducing LGCI	Low
Bao et al. (2020) [67]	Narrative Review	2020	Literature revision regarding critical effects displayed by fatty acids on	-	Potential therapeutic implications of fatty acids in multiple bone disorders	-	Fatty acids is potential therapeutic and nutritional agents for the treatment and prevention of metabolic bone diseases	Low

			bone metabolism					
Garcia-Martinez et al. (2014) [70]	Sistematic Review	December 2012	databases: PubMed, Scopus, Medline, and Cochrane.	37 articles	Incidence of osteoporosis in Mediterranea diet	-	Mediterranean diet may have a protective effect on bone health and the potentially responsible for this effect is olive oil	High
Garcia-Gallivan et al. (2016) [71]	Randomized controlled trial	8.9 years	FFQ	870	Consumption of total olive oil and its varieties on the risk of osteoporosis-related fractures	In the higher tertile of EVOO consumption (56.5 g/day) was relatively 46% lower (P = 0.050) than the reference tertile.	Adequate consumption of EVOO oil prevents osteoporosis-related fractures	High
Roncero-Martin et al. (2018) [72]	Cross-sectional study	2010-2017	FFQ, DXA, computed tomography scans	523 women (age 23-81)	Association between olive oil intake and cortical and trabecular bone microarchitecture.	Women with a higher dietary intake of olive oil (>18.32 g/day) had significantly higher total, trabecular and cortical bone density compared with those with a lower intake of olive oil (≤18.32 g/day).	Dietary intake of olive oil is positively associated with a better volumetric bone mineral density (vBMD)	Moderate
