# Supplementary material for: Nutrition, Physical Activity, and Dietary Supplementation to Prevent Bone Mineral Density Loss: A Food Pyramid
Source: Nutrients. 2021 Dec 24;14(1):74. doi: 10.3390/nu14010074 (PMC8746518; doi:10.3390/nu14010074)
Supplement: Supplementary file 1 [file nutrients-14-00074-s001.zip › nutrients-1519822-supplementary/Table S4b. Lipids supplementation.pdf]

Author	Type of study	Study period	Supplementation	Subjects	End point	Results	Conclusion	Strength of evidence
Van Papendorp et al. (1995) [68]	Clinical trial	16 weeks	n 10 = 4 g fish oil (containing EPA and DHA); n 10 = 4 g olive oil; n 10= 4 g evening primrose oil; N 10 = 4 g mixture fish oil/ebening primrose oil	40 old women (mean age 80±4)	Role essential fatty acid (EFA) supplementation in the improvement of osteoporosis	Increase in serum calcium and in urinary calcium clearance (p< 0.05) in fish oil group Osteocalcin increased in fish oil and in fish oil/evening primrose oil groups p<O.O51)	Supplementation with fish oil (or a mixture of fish oil and primrose oil) may enhance bone formation	Moderate
Kruger et al. (1998) [69]	Clinical trial	18 months	-6 g gamma-linolenic acid (GLA)+EPA<br><br>- 6 g coconut oil	65 women (mean age 79.5)	Interactions between calcium and GLA+EPA	Lumbar spine density remained the same in the treatment group, but decreased 3.2% in the placebo group. Femoral bone density increased 1.3% in the treatment group, but decreased 2.1% in the placebo group.	GLA and EPA have beneficial effects on bone	Moderate
