# Supplementary material for: Nutrition, Physical Activity, and Dietary Supplementation to Prevent Bone Mineral Density Loss: A Food Pyramid
Source: Nutrients. 2021 Dec 24;14(1):74. doi: 10.3390/nu14010074 (PMC8746518; doi:10.3390/nu14010074)
Supplement: Supplementary file 1 [file nutrients-14-00074-s001.zip › nutrients-1519822-supplementary/Table S5a. Protein intake.pdf]

Author	Type of study	Study period	Methods	Subjects	End point	Results	Conclusion	Strenght of evidence
Margen et al. (1974) [73]	Narrative review	-	Analysis of the diet with variable protein and amino acid content, collection of 24-h urine samples	26 healthy male volunteers, aged from 20 to 32 y	The effect of protein intake on urinary calcium excretion	A markedly positive correlation between protein ingestion and calciuria	Varying protein intake results in approximately an 800% increase in calcium excretion, irrespective of calcium intake	Low
Schuette et al. (1980) [74]	Longitudinal study	30 days	A controlled diet in protein intake was administered in two phases: first low-protein then high protein. multiple Blood samples were collected	5 men (age 44-86 y) and 6 women (age 65-79 y)	Correlation between protein intake, urinary calcium and calcium balance. Mechanisms involved in protein-induced hypercalciuria.	An increase in protein intake from about 47 to 112 g while maintaining calcium, magnesium and phosphorus intakes constant caused an increase in urinary calcium and a decrease in calcium retention.	Protein-induced hypercalciuria is due to an increase in glomerular filtration rate and a decrease in fractional renal tubular reabsorption of calcium.	Moderate
Breslau et al. (1988) [75]	Longitudinal study	Three consecutive phases of 12-days.	- Constant diet: calcium 400 mg or 10.0 mmol/day, phosphorus 1000 mg or 32.3 mmol/day, sodium 100 mmol/day and total protein 75 g/day (as animal protein during one phase and as soy-based	15 adults (8 women and 7 men) Age: 26-46 y	Possible effects of different types of dietary proteins on calcium metabolism and on the propensity to form kidney stones	Urinary calcium excretion increased from 103 ± 15 mg/day on the vegetarian diet to 150 ± 13 mg/day on the animal protein diet (P < 0.02). Oxalate excretion lower than during the	A diet rich in animal proteins appears to pose a risk for the formation of uric acid stones, but not for calcium stones	Moderate

			vegetable protein with eggs during a second phase<br>- Blood and 24-h urine samples			vegetarian diet (26 ± 1 mg/day vs. 39 ± 2 mg/day; P < 0.02)		
Hu et al. (1993) [76]	Cross-sectional study	1993	- Urine and blood samples<br>- Food diary (3 days) for evaluated dietary intakes	764 women aged 35-75 y	Correlation of dietary components and urinary excretion of acids and calcium	Urinary excretions of calcium and acids are correlated positively with intakes of animal and nondairy protein but are correlated negatively with vegetal-protein intakes	Calcium excretion is determined by the acid-base status of the diet	Moderate
Feskanich et al. (1996) [77]	Cohort study	1976-1986	- Mailed questionnaire for anthropometric measures and presence oh hip or forearm fractures<br>- Semiquantitative food frequency questionnaire (FFQ) for estimated dietary intakes	85900 women aged 35-59 y	The relation between dietary protein and the incidence of adult bone fractures	Proteins are associated with an increased risk of forearm fracture (relative risk (RR) = 1.22, 95% confidence interval (CO 1.04-1.43, p = 0.01) for women who consume more than 95 g/day compared with less than 68 g/day. Women who consume five or more servings of red meat per week also have a significantly increased risk of forearm fracture (RR = 1.23, 95% Cl 1.01-1.50) .	Higher protein consumption increases the risk of osteoporotic forearm fractures	Moderate
Dolan et al. (2019) [78]	Narrative review	2019	Revision of the literature that take in consideration the	About 350000	Summarize the potential	It's time to abandon the long-held belief	Protein is an essential	Low

			relation between proteins and bone	subjects (men and women)	mechanisms that may lead to either a positive or a negative influence of protein on bone	that higher protein intakes lead to bone demineralisation, particularly in healthy individuals who have an adequate calcium intake	nutrient for bone health	
Kerstetter et al. (1997) [79]	Longitudinal study	2 weeks of an adjustment diet, followed by 4 days of experimental diet and 3 days of an ad libitum diet	- 2-weeks of adjustment period: 1 g protein/kg,<br>- 4 days of experimental diet: protein intake low (0.7 g/kg), medium (1.0 g/kg), or high (2.1 g/kg).<br>- Blood and 24-h urine samples	16 healthy women with mean age 26.7 ±1.3 y	The effect of three amounts of dietary protein on mineral metabolism and the PTH-l-α-hydroxylase axis	Urinary calcium decreases significantly with the low-protein diet and increases significantly with the high-protein diet. Striking elevations in serum PTH and calcitriol in subjects consuming the low-protein diet	A low protein intake may be associated with reduced bone turnover and intestinal calcium absorption, a medium protein intake does not affect these processes, and a high protein intake may suppress the PTH-l-α -hydroxylase axis by enhancing bone resorption, calcium absorption, or both	Moderate
Kerstetter et al. (2000) [80]	Longitudinal study	2 weeks of an adjustment diet, followed by 4 days of experimental diet and 3 days of an ad libitum	- 2-weeks of adjustment period: 1 g protein/kg,<br>- 4 days of experimental diet: protein intake 0.7, 0.8, 0.9, and 1.0 g/kg<br>- Blood and 24-h urine samples	8 women, mean age of 23.1 ± 2.3 y	The effect of graded intakes of dietary protein (0.7, 0.8, 0.9, and 1.0 g/kg) on calcium homeostasis	Elevations in PTH develops by day 4 of the diets containing 0.7 and 0.8 g protein/kg but not during the diets containing 0.9 or 1.0 g protein/kg. Mean	In young healthy women consuming a well-balanced diet, the current recommended dietary allowance for protein (0.8 g/kg) results in	Moderate

		diet				24-h urinary calcium is 3.29 ± 0.35 mmol with the diet containing 0.7 g protein/kg and 3.54 ± 0.46 mmol with the diet containing 1.0 g protein/kg	short-term perturbations in calcium homeostasis	
Giannini et al. (1999) [81]	Longitudinal study	2 weeks	- diet controlled only for calcium and sodium intake<br>- validated dietetic<br>- questionnaire<br>- Blood and 24-h urine samples	18 (10 men and 8 women, age: 45.6 ± 12.3 y)	Effects of moderate protein restriction in hypercalciuric patients	Urinary excretion of urea falls after the diet ($P < 0.001$). Urinary calcium ($P < 0.001$), uric acid ($P < 0.005$), oxalate ($P < 0.01$), and hydroxyproline ($P < 0.01$) decrease after protein restriction	In hypercalciuric patients, moderate protein restriction decreases calcium excretion, mainly through a reduction in bone resorption and renal calcium loss	Moderate
Heaney et al. (2008) [82]	Narrative review	2008	Revision of the literature that take in consideration the dietary and physiologic factors that affect bone health	about 3000 subjects (men and women)	The relation of protein in the diet, the protein source, calcium intake, weight loss, and the acid/base balance of the diet with bone health	Higher protein diets are associated with greater bone mass and fewer fractures when calcium intake is adequate	Calcium and protein intake interact constructively to affect bone health	Low
Mangano et al. (2014) [83]	Narrative review	2013 - 2014 (18 months)	Relevant data from epidemiological studies, intervention studies and meta-analysis	20951 (men and women)	The association between dietary protein, calcium and bone health	Dietary protein may positively impact bone health by increasing muscle mass, increasing calcium absorption, suppressing parathyroid hormone, and	The positive effects of protein intake on bone health may only be beneficial under conditions of adequate calcium intake	Low

						augmenting insulin like growth factor 1 production		
Darling et al. (2009) [84]	Systematic review and meta-analysis	2009	The MEDLINE (January 1966 to September 2007) and EMBASE (1974 to July 2008) databases are electronically searched for all relevant studies of healthy adults	61 studies for the systematic review, of which 28 included in the meta-analysis (466789 subjects, men and women)	The relation between protein and bone health in healthy human adults	In cross-sectional studies, all pooled r values for the relation between protein intake and BMD are significant and positive. The meta-analysis of randomized placebo-controlled trials indicates a significant positive influence of all protein supplementation on lumbar spine BMD but shows no association with relative risk of hip fractures	A small positive effect of protein supplementation on lumbar spine BMD in randomized placebo-controlled trials supports the positive association between protein intake and bone health found in cross-sectional studies	High
Shams-White et al. (2017) [85]	Systematic review and meta-analysis	October 2016	5 databases: Ovid Medline (1946 to 4 October 2016), Cochrane Central Register of Controlled Trials (1991 to 31 October 2016), Scopus (+ EMBASE; 1974 to 31 October 2016), Web of Science (1864 to 31 October 2016), and Global Health (1910 to 31 October 2016)	16 RCTs and 20 prospective cohort studies are included in the systematic review	The effects of dietary protein intake alone and with calcium with or without vitamin D on bone health measures in adults.	An higher protein intake may have a protective effect on lumbar spine (LS) BMD compared with lower protein intake but no effect on total hip (TH), femoral neck (FN), or total body BMD or bone biomarkers	Only the LS shows moderate evidence to support benefits of higher protein intake	High
Groenendijk et al. (2019) [86]	Systematic review and meta-analysis	29 October 2018	3 databases: CENTRAL, MEDLINE, EMBASE	12 cohort studies and 1 RCT, of	The impact of a dietary protein intake above the Recommended Dietary	A positive trend between higher protein intakes and	A protein intake above the current RDA	High

				which 4 included in the meta-analysis. Men and women aged ≥ 65 y	Allowance (RDA) of 0.8 g/kg body weight/day from any source on BMD/Bone Mineral Content (BMC), bone turnover markers, and fracture risk in older adults compared to a lower dietary protein intake	higher femoral neck and total hip BMD. Higher protein intakes results in a significant decrease in hip fractures (pooled hazard ratio: 0.89; 95% confidence interval: 0.84, 0.94)	may reduce hip fracture risk and may play a beneficial role in BMD maintenance and loss in older adults	
Wallace et al. (2017) [87]	Systematic review and meta-analysis	11 April 2017	3 databases: PubMed, Ovid Medline, and Agricola	29 articles included (16 randomized RCTs and 13 prospective cohort studies, of which 4 included in the meta-analysis. 274169 adults (men and women)	A possible relationship between dietary protein intake above the current RDA of 0.8 g/kg/d on fractures, BMD/BMC, and markers of bone turnover	High vs low protein intakes result in a statistically significant 16% decrease in hip fractures (standardized mean difference (SMD) = 0.84, 95% confidence interval, 0.73, 0.95; I2 =36.8%).	Protein intakes above the current RDA may have some beneficial role in preventing hip fractures and BMD loss	High
