# Supplementary material for: Nutrition, Physical Activity, and Dietary Supplementation to Prevent Bone Mineral Density Loss: A Food Pyramid
Source: Nutrients. 2021 Dec 24;14(1):74. doi: 10.3390/nu14010074 (PMC8746518; doi:10.3390/nu14010074)
Supplement: Supplementary file 1 [file nutrients-14-00074-s001.zip › nutrients-1519822-supplementary/Table S5b. Protein supplementation.pdf]

Author	Type of study	Study period	Supplementation	Subjects	End point
Zhu et al. (2011) [91]	Randomized, double-blind, placebo-controlled trial	2 years	Protein group: 250 ml of skim milk-based high-protein supplement drink reconstituted with cold water from a powder that provided 30 g of protein, 600 mg of calcium, and 3.2 kJ/ml of energy	196 women (protein group: n = 101, mean age 74.2 ± 2.8 y; placebo group: n = 95, mean age 74.3 ± 2.6 y)	The effect of protein supplementation on BMD and strength, and the effect on calcium excretion and serum IGF-1 of older postmenopausal women
Kerstetter et al. (2015) [92]	Randomized, double-blind, placebo-controlled trial	18 months	45-g whey protein	208 subjects, men and women (protein group: n = 106, mean age 69.9 ± 6.1 y; placebo group: n = 102, mean age: 70.5 ± 6.4 y)	The impact of a moderately high protein diet on BMD
Hettiarachchi et al. (2019) [93]	Randomized cross-over trial	2 days	A calcium-fortified milk-based protein supplement (MBPM): 55g powder reconstituted with 220 ml of water	16 postmenopausal women with mean age 64.7 ± 3.3 y	The effect of bedtime ingestion of a MBPM or maltodextrin (CON) on acute (0–4 h) blood and 24-h urinary change in biomarkers of bone remodeling in postmenopausal women with osteopeni[illegible]
