# Supplementary material for: Nutrition, Physical Activity, and Dietary Supplementation to Prevent Bone Mineral Density Loss: A Food Pyramid
Source: Nutrients. 2021 Dec 24;14(1):74. doi: 10.3390/nu14010074 (PMC8746518; doi:10.3390/nu14010074)
Supplement: Supplementary file 1 [file nutrients-14-00074-s001.zip › nutrients-1519822-supplementary/Table S6a. Vitamin A intake.pdf]

Author	Type of study	Study period	Methods	Subjects	End point	Results	Conclusion	Strength of evidence
Crandall et al. (2004) [97]	Narrative Review	24 hours – 30 years	A MEDLINE search with keywords retinol, β-carotene, and osteoporosis.	119057 subjects	Circulating RBP levels, BMC (distal forearm, lumbar spine, femoral neck), hip fractures, serum calcium and PTH, urine CrossLaps, calcium/creatinine, skeletal changes by skeletal radiographs, serum BSAP, NTX, OC.	6 studies suggesting possible benefit of vit A on osteoporosis, 11 studies suggesting adverse effects, 3 studies suggesting null effect.	It's not possible to set a specific level of retinol intake above which bone health is compromised.	Low
Penniston et al. (2006) [99]	Narrative Review	Follow-up of 4 – 30 years	//	110320 subjects	Hip fractures and all fractures, BMD.	Slightly greater risk for hip fracture (RR 1.18) with supplement use; no dose- response relation observed. Serum retinol: highest vs lowest quintile, RR 1.64 for any fracture and 2.47 for hip fracture; diet: retinol ≥ 1500 µg/d associated with doubled risk of any fracture. Lower BMD associated with retinol intake up to peak intake of 2000–2800 IU (effect more pronounced in women). Retinol intake ≥ 2000 versus < 500 µg/d nearly doubled the rate of hip fracture.	Intakes much lower than 10 times the RDA, the amount conventionally thought to lead to toxicity, are needed to increase risk for osteoporosis—ie, ≈ 2X RDA.	Low
Jakcson et al. (2005) [101]	Narrative Review	64 months – 30 years	Literature search using MEDLINE (1966 - March	112100 women, 2322 men	Hip and non-hip fractures.	Retinol intake > 1500 µg/day and mean vitamin intake ≥ 3000 µg/day associated with	Consumption of large amounts of vit. A may be associated with decreased bone mineral density	Low

			2005)			significant increase in risk of hip fracture; vitamin A serum concentrations in the highest quintile (≥ 2.56 µmol/L) associated with a significant increase in risk of hip fracture (HR 2.1; 95% CI 1.2 to 3.6); vitamin A serum concentrations in lowest quintile (≤ 1.61 µmol/L) associated with a significant increase in hip fracture risk (HR 1.9; 95% CI 1.1 to 3.3). In two studies no associations between vitamin A and retinol intake and fracture risk.	and increased fracture risk.	
Yee et al. (2021) [94]	Narrative Review	//	//	269990 subjects	BMD at various sites; hip fracture risk; all fractures risk; total body BMC; serum calcium; ALP, NTx and OCN.	8 studies showed a protective effect of vitamin A, 10 showed negative effects, while 9 found no association between vitamin A intake and BMD or risk of fractures.	In humans the direct relationship between vitamin A and poor bone health was more pronounced in individuals with obesity or vitamin D deficiency; however, adequate vitamin A intake through food was shown to maintain healthy bones. Meanwhile, provitamin A (carotene and β-cryptoxanthin) may also protect bone.	Low
