# Supplementary material for: Nutrition, Physical Activity, and Dietary Supplementation to Prevent Bone Mineral Density Loss: A Food Pyramid
Source: Nutrients. 2021 Dec 24;14(1):74. doi: 10.3390/nu14010074 (PMC8746518; doi:10.3390/nu14010074)
Supplement: Supplementary file 1 [file nutrients-14-00074-s001.zip › nutrients-1519822-supplementary/Table S6b. Vitamin A supplementation.pdf]

Author	Type of study	Study period	Supplementation	Subjects (age, sex, number…)	End point	Results (numbers)	Conclusion (descriptive)	Strength of evidence
Johansson et al. (2001) [96]	RCT (double-blind crossover study)	24 hours	15 mg of retinyl palmitate; 2 µg of $1,25(OH)_2D_3$; 15 mg of retinyl palmitate *plus* 2 µg of $1,25(OH)_2D_3$	9 healthy subjects (4 M – 5 F) aged 24–41 years (mean age: 28 years)	Serum $1,25(OH)_2D_3$, S-retinyl esters, S-calcium, S-albumin, S-parathyroid hormone (PTH), the degradation product of C-telopeptide of type I collagen (S-CrossLaps); urine calcium (U-calcium) and U-creatinine.	S-$1,25(OH)_2D_3$ and retinyl esters increased (1.7-fold and 8.3-fold, respectively; $p<0.01$). S-calcium increased (2.3%; $p<0.01$) and S-PTH decreased (232%; $p<0.05$) after $1,25(OH)_2D_3$ intake. In contrast, retinyl palmitate intake resulted in a significant decrease in S-calcium when taken alone (21.0%; $p<0.05$) and diminished the calcium response to $1,25(OH)_2D_3$ after the combined intake (1.4%; $p<0.01$). No significant changes in S-CrossLaps, or U-calcium/creatinine levels.	An intake of vitamin A corresponding to about one serving of liver antagonizes the rapid intestinal calcium response to physiological levels of vitamin D.	High
Crandall et al. (2004) [97]	Narrative review	24 hours – 30 years	Vitamin A, retinoyl palmitate, calcium and vitamin D at different dosages and in various combinations.	119057 subjects	Circulating RBP levels, BMC (distal forearm, lumbar spine, femoral neck), hip fractures, serum calcium and PTH, urine	6 studies suggesting possible benefit of vit A on osteoporosis, 11 studies suggesting adverse effects, 3 studies suggesting null effect.	It's not possible to set a specific level of retinol intake above which bone health is compromised; vit A supplements should not be used with the express goal of improving bone health.	Low

					CrossLaps, calcium/creatinine, skeletal changes by skeletal radiographs, serum BSAP, NTX, OC.			
Yee et al. (2021) [94]	Narrative review	1986; 1993 - 1998	Vitamin A and retinol intake from supplement.	189920 postmenopausal women aged 34 – 79 years	Fracture risk	Vitamin A [RR = 1.22 (95% CI 0.98–1.52)] and retinol [RR = 1.24 (95% CI 0.96–1.59)] supplement users had higher hip fracture risk compared to non-users. Vitamin A [RR = 1.48 (95% CI 1.05–2.07)] and retinol [HR = 1.89 (95% CI 1.33–2.68)] intake from food plus supplement were positively associated with risk of hip fracture. Total vitamin A [HR = 1.19 (95% Cl 1.04–1.34)] and retinol intake [HR = 1.15 (95% CI 1.03–1.29)] was positively associated with fracture risk.	High total vitamin A and retinol intakes were associated with fracture risk with low vitamin D intake.	Low
