# Supplementary material for: Nutrition, Physical Activity, and Dietary Supplementation to Prevent Bone Mineral Density Loss: A Food Pyramid
Source: Nutrients. 2021 Dec 24;14(1):74. doi: 10.3390/nu14010074 (PMC8746518; doi:10.3390/nu14010074)
Supplement: Supplementary file 1 [file nutrients-14-00074-s001.zip › nutrients-1519822-supplementary/Table S7a. Vitamin D intake.pdf]

Author	Type of study	Study period	Methods	Subjects	End point	Results	Conclusion	Strenght of evidence
Van der Mei et al. (2007) [106]	Narrative review	Data from three published studies and the TasOAC study	Methodology of Engelsen et al. (2005) to estimate maximum daily duration of vitamin D synthesis in human skin.	< 60years	Comparison of vitamin D status [25-hydroxyvitamin D; 25(OH)D] in people < 60 years of age using data from cross-sectional studies of three regions across Australia	The prevalence of vitamin D insufficiency (≤ 50 nmol/L) in women in winter/spring was 40.5% in southeast Queensland, 37.4% in the Geelong region, and 67.3% in Tasmania. Season, simulated maximum daily duration of vitamin D synthesis, and vitamin D effective daily dose each explained around 14% of the variation in 25(OH)D.	Vitamin D insufficiency is common over a wide latitude range in Australia. Current sun exposure guidelines do not seem to fully prevent vitamin D insufficiency.	Low
Freisling et al. (2010) [107]	Cross-sectional study	/	- Standardized 24-h dietary recall<br><br>- Intake of 25 nutrients (excluding intake from dietary supplements) estimated using a standardized nutrient database.	36,034 persons aged 35–74	The diversity of nutrient patterns in the European Prospective Investigation into Cancer and Nutrition (EPIC) study at population level as a starting point for future nutrient pattern analyses and their associations with chronic diseases in multi-center studies.	For all countries combined, the daily mean vitamin D intake was reported as 4.8 µg/day for men and 3.3 µg/day for women.	Various national and European strategies have been suggested to facilitate the achievement of adequate vitamin D status in European populations.	Moderate
