# Supplementary material for: Nutrition, Physical Activity, and Dietary Supplementation to Prevent Bone Mineral Density Loss: A Food Pyramid
Source: Nutrients. 2021 Dec 24;14(1):74. doi: 10.3390/nu14010074 (PMC8746518; doi:10.3390/nu14010074)
Supplement: Supplementary file 1 [file nutrients-14-00074-s001.zip › nutrients-1519822-supplementary/Table S7b. Vitamin D supplementation.pdf]

Author	Type of study	Study period	Supplementation	Subjects	End point	Results	Conclusion	Strenght of evidence
Hunter et al. (2000) [113]	Clinical Trial	2 years	For each twin pair:<br>• one was randomized to 800 IU cholecalciferol/day for 2 years<br>• the other was randomized to placebo	79 monozygotic (MZ) postmenopausal twin pairs	The effect of vitamin D3 supplementation on bone density and bone metabolism in young postmenopausal women.	Over the first 6 months, serum 25(OH)D increased in the treatment group by 57% and this was significantly different from the placebo group, which increased by 15%. At 24 months, differences in the mean concentration of vitamin D from baseline were maintained; the treatment group had increased 47% and the placebo group had increased 12%.	Vitamin D supplementation on its own cannot be recommended routinely as an osteoporosis prevention for healthy postmenopausal women with normal vitamin D levels under the age of 70 years.	High
Bischoff-Ferrari et al. (2009) [114]	Meta-analysis of Randomized Controlled Trials	RCTs that studied oral vitamin D supplementation or ergocalciferol minimum follow-up of 1 year	• Dose ≤ 400 IU/d<br><br>• Dose of 482 to 770 IU/d	Age ≥ 65 years	Test the efficacy of oral supplemental vitamin D in the prevention of non-vertebral and hip fractures among older individuals	No fracture reduction was observed for a received dose of 400 IU/d or less, whereas a higher received dose of 482 to 770 IU/d of supplemental vitamin D reduced nonvertebral fractures by 20% and hip fractures by 18%.	Nonvertebral fracture prevention with vitamin D is dose dependent, and a higher dose should reduce fractures by at least 20% for individuals aged 65 years or older.	High
Weaver et al. (2016) [112]	Meta-analysis of Randomized Controlled Trials	PubMed and Medline literature search was conducted for the period from July 1, 2011 through July 31, 2015.<br><br>Excluded short-term (<1 month) RCTs	RCTs of generally healthy adults that compared vitamin D supplementation plus calcium against no supplementation or placebo.	Healthy adults (<20 % of study participants had major chronic diseases such as diabetes or cardiovascular disease, at baseline)	A meta-analysis of randomized controlled trials of calcium plus vitamin D supplementation and fracture prevention in adults.	Meta-analysis showed a significant 15 % reduced risk of total fractures (summary relative risk estimate [SRRE], 0.85; 95 % confidence interval [CI], 0.73–0.98) and a 30 % reduced risk of hip fractures (SRRE, 0.70; 95 % CI, 0.56–0.87).	This meta-analysis of RCTs supports the use of calcium plus vitamin D supplements as an intervention for fracture risk reduction in both community-dwelling and institutionalized middle-aged to older adults.	High
Bonjour et al. (2009) [118]	Clinial Trial	1 month	Consumption of soft plain cheese made of semi-skimmed milk which was fortified by both vitamin D3 (+1·25 μg/100 g) and milk Ca thus achieving a total Ca content of 151 mg/100 g as compared with 90–120 mg/100 g for standard fresh cheese.	37 Women aged 65 years or older<br><br>Ca intake lower than 700 mg/d<br><br>Sun exposure of uncovered arms limited to less than 20 min/d<br><br>Serum 25-hydroxyvitamin D (25(OH)D) ≥ 4 ng/ml<br><br>Serum PTH ≥ 46 ng/l and ≤ 150 ng/l<br><br>Creatinine clearance normal or moderately reduced with value ≥ 30 ml/min<br><br>no dislike for	Explore in elderly women whether a vitamin D and Ca-fortified dairy product providing about 17–25 % of the recommended intakes in vitamin D, Ca and proteins would reduce secondary hyperparathyroidism and bone remodelling in a way that may attenuate age-related bone loss in the long term.	Mean serum changes were: 25-hydroyvitamin D, +14·5 % (P = 0·0051); parathyroid hormone (PTH), − 12·3 % (P = 0·0011); CTX, − 7·5 % (P = 0·01); tartrate-resistant acid phosphatase isoform 5b (TRAP 5b), − 9·9 % (P < 0·0001); albumin, +6·2 % (P < 0·0001); insulin-like growth factor-I (IGF-I),+16·9 % (P < 0·0001); osteocalcin, +8·3 % (P = 0·0166); amino-terminal propeptide of type 1 procollagen (P1NP),+19·3 % (P = 0·0031).	Fortified soft plain cheese consumed by elderly women with vitamin D insufficiency can reduce bone resorption markers by positively influencing Ca and protein economy, as expressed by decreased PTH and increased IGF-I, respectively.	High

				dairy products<br><br>Mini Nutritional Assessment with a score ≥ 21				
Reid et al. (2014) [115]	Systematic review and meta-analysis	from inception to July 8, 2012	<800IU/die<br><br>>800IU/die	adults<br><br>(average age >20 years) without other metabolic bone diseases	We investigated whether vitamin D supplementation aff ects bone mineral density.	Results of our meta-analysis showed a small benefi t at the femoral neck (weighted mean diff erence 0·8%, 95% CI 0·2–1·4) with heterogeneity among trials (I^2=67%, p<0·00027). No eff ect at any other site was reported, including the total hip	Continuing widespread use of vitamin D for osteoporosis prevention in community-dwelling adults without specific risk factors for vitamin D deficiency seems to be inappropriate.	High
Yao et al. (2019) [117]	Systematic review and meta-analysis	December 2018, July 2019	• 800 IU/d vitamin D; 1200 mg/d calcium<br><br>• 100 000 IU/4 mo<br><br>• 300 000 IU/y<br><br>• 800 IU/d	Observational studies involving at least 200 fracture cases and RCTs enrolling at least 500 participants and reporting at least 10 incident fractures were included.	To assess the risks of fracture associated with differences in concentrations of 25-hydroxyvitamin D (25[OH]D) in observational studies and the risks of fracture associated with supplementation with vitamin D alone or in combination with calcium in RCTs.	In a meta-analysis of 11 observational studies each increase of 10.0 ng/mL (ie, 25 nmol/L) in 25 (OH)D concentration was associated with an adjusted RR for any fracture of 0.93 (95%CI, 0.89-0.96) and an adjusted RR for hip fracture of 0.80 (95%CI, 0.75-0.86). A meta-analysis of 6 RCTs of combined supplementation with vitamin D (daily doses of 400-800 IU, yielding a median difference in 25[OH]D concentration of 9.2 ng/mL) and calcium (daily doses of 1000-1200mg) found a 6%reduced risk of any fracture (RR, 0.94; 95%CI, 0.89-0.99) and a 16%reduced risk of hip fracture (RR, 0.84; 95%CI, 0.72-0.97).	This systematic review and meta-analysis of randomized clinical<br>trials of vitamin D alone<br>showed no significant association with<br>risk of any fracture or of hip fracture. In<br>contrast, daily supplementation with both vitamin D and calcium (6 randomized clinical trials with 49 282<br>participants) was associated with a 16%<br>reduced risk of hip fracture.	High
Zhao et al. (2017) [116]	Systematic Review and Meta-analysis	from July 16, 2012, to July 16, 2017.	• Calcium supplementation (≥1 or <1 g/d)<br><br>• Vitamin D supplementation (≥800 IU/d; <800 IU/d; intermittent high-dose given as once every year; intermittent high-dose given as other frequencies, including once every 3 or 4months and once every 1week or month)<br><br>• Combined calcium and vitamin D supplementation	Community-dwelling adults older than 50 years.	Whether calcium, vitamin D, or combined calcium and<br>vitamin D supplements are associated with a lower fracture incidence in community-dwelling older adults.	No significant association of calcium or vitamin D with risk of hip fracture.	The use of supplements that included calcium, vitamin D, or both compared with placebo or no treatment was not associated with a lower risk of fractures among community-dwelling older adults..	High
