# Supplementary material for: Nutrition, Physical Activity, and Dietary Supplementation to Prevent Bone Mineral Density Loss: A Food Pyramid
Source: Nutrients. 2021 Dec 24;14(1):74. doi: 10.3390/nu14010074 (PMC8746518; doi:10.3390/nu14010074)
Supplement: Supplementary file 1 [file nutrients-14-00074-s001.zip › nutrients-1519822-supplementary/Table S8. Vitamin E intake.pdf]

| Author | Type of study | Study period | Methods | Subjects | End point | Results | Conclusion | Strenght of evidence |
|---|---|---|---|---|---|---|---|---|
| Guralp et al. (2014) [119] | Mini-narrative review | Literature between february 2014 and august 2014 | Literature search using Scopus and Pubmed | 19 articles focused on effects of vitamin E on bone health | Effects of vitamin E on BMD and fracture risk in perimenopausal women. | - | Current evidence does not provide a causal relationship between vit. E and osteoporosis and hip fracture risk. | Low |
| Shuid et al. (2014) [120] | Systematic review | studies published between 2005 and 2015 | Medline and Scopus database were searched | A total of 8 studies of which: 5 on animals, 1 in vitro and 2 on humans | The therapeutic effect of vitamin E in preventing bone loss | - | Vitamin E, in particular tocotrienol, has an important anti-inflammatory and immunomodulatory role related to bone metabolism. Vitamin E exerts its anti-osteoporotic action | High |
| Zhou et al. (2020) [121] | Meta-analysis | Studies published throughout October 2019 | PubMed, EMBASE AND Cochrane Library electronic database studies | 13 prospective cohort studies were selected for a total of 384,464 individuals | Reliable estimates for dietary antioxidant vitamin (vit. A, C and E) intake and their effect on fracture risk at various sites. | Increased antioxidant vitamin intake was associated with a reduced fracture risk (RR: 0.92; 95% CI: 0.86-0.98; P = .015) | Fracture risk at any site is significantly reduced with increased antioxidant vitamin intake, especially vitamin E intake and in men. | High |
| Odai e al. (2019) [122] | Cross-sectional retrospective study | From january 2009 to august 2017 | DXA and brief-type self-administered diet history questionnaire (BDHQ) | 157 women aged 38–76, divided into premenopausal (n=46) and postmenopausal (n=111) groups. | The relationship between the consumption of various nutrients (43) and BMD in middle-aged women | The average age of the participants was 54.5 +- 7.0 years, and BMI was 21.7 +- 3.5 kg/m2. Their BMD and BMD Z-scores were 1.044 +- 0.167 g/cm2 and 0.15 +- 1.26, respectively. Correlation between daily intake of alpha-tocopherol and BMD Z-score, R = 0.33, p = 0.027) | The dietary intake of vitamin E was shown to be positively associated with BMD in premenopausal Japanese women. An increase in the dietary consumption of VE could help maintain the bone mass in premenopausal women. | Moderate |
| Michaëlsson et al. (2021) [123] | Mendelian randomization study | 2021 | Mendelian randomization investigation; heel ultrasound and fractures (from hospital records and by self-reports) | 3 genome-wide association studies comprising 7781 indivisuals of European ancestry.<br><br>426,824 participants and fracture (53,184 cases and 373,611 non-case) were taken from GWASs based on data from UK Biobank. | The associations of circulating alpha-tocopherol with BMD | A predicted one-standard-deviation increase of circulating alpha-tocopherol was associated with 0.07 g / cm2 in BMD, which corresponds to a> 10% higher BMD | Increasing circulating alpha-tocopherol is associated with higher BMD. | High |
