# Supplementary material for: Nutrition, Physical Activity, and Dietary Supplementation to Prevent Bone Mineral Density Loss: A Food Pyramid
Source: Nutrients. 2021 Dec 24;14(1):74. doi: 10.3390/nu14010074 (PMC8746518; doi:10.3390/nu14010074)
Supplement: Supplementary file 1 [file nutrients-14-00074-s001.zip › nutrients-1519822-supplementary/Table S9a. Vitamin K intake.pdf]

Author	Type of study	Study period	Methods	Subjects	End point	Results	Conclusion	Strenght of evidence
Feskanich et al. (1999) [128]	Cohort study	1984-1994	- FFQ<br>- Weight requested on all biennial questionnaires.<br>-BMI and physical activity categorized into quintiles for analysis.	72,327 women aged 38–63 years	High intakes of vitamin K are associated with a lower risk of hip fracture in women.	Women in quintiles 2–5 of vitamin K intake had a significantly lower age-adjusted relative risk (RR: 0.70; 95% CI: 0.53, 0.93) of hip fracture than women in the lowest quintile (< 109 mg/d).	High intakes of vitamin K can lower serum concentrations of Undercarboxylated osteocalcin, positively associated with risk of hip fractures. Low intakes of vitamin K may increase the risk of hip fracture in women.	Moderate
Palermo et al. (2017) [124]	Systematic review	2017	Investigating the different forms of Vitamin K and their effect on bone metabolism	-	The impact of vitamin K on bone health with particular interest in bone remodeling, mineral density and fragility fractures	Low concentrations of Vitamin K seem to be associated with an increased risk of fracture in different populations	In young and elderly women, low Vitamin K intake seems to be associated with bone deterioration.	High
Akbari et al. (2018) [125]	Narrative Review	2018	-	-	Comprehensively outline the preclinical studies on the properties of vitamin K and its effects on the bone metabolism.	Vitamin K affects the proliferation and differentiation of osteoblasts.	Vitamins K2, especially MK-4, promotes bone formation by stimulating the differentiation of the osteoblast, regulating the mineralization of the extracellular matrix.	Low
Iwanoto et al. (2014) [126]	Narrative Review	2014	RCTs that investigated the effect of menatetrenone on BMD, measured by DXA and fracture incidence in postmenopausal women with osteoporosis	-	Clarify the effect of menatetrenone on the skeleton in postmenopausal women with osteoporosis.	Menatetrenone monotherapy decreased serum undercarboxylated osteocalcin (ucOC) concentrations, modestly increased lumbar spine BMD, and reduced the incidence of fractures (mainly vertebral fracture).	A positive evidence for the effects of menatetrenone monotherapy on fracture incidence in postmenopausal women with osteoporosis.	Low
Hamidi et	Narrative	2013	-	-	The different	Low circulating vitamin	A diet high in vitamin K is	Low

al. (2013) [127]	Review				forms and sources of Vitamin K and its effect on BMD and fractures.	K1, low vitamin K1 intake, low vitamin K2 (MK-7) intake, and high serum levels of undercarboxylated osteocalcin have been associated with increased risk of hip fractures.	associated with a lower risk of hip fractures in aging men and women.	
Shah et al. (2014) [129]	Systematic review	RCT studies from 2000 to 2010	PubMed search	-	The role of vitamin K in bone health in older adults and the clinical implications	An increased risk of hip fracture in the nurses who consumed the lowest quintile of dietary vitamin K (< 109 μg/d) compared with the nurses with the highest quintile of dietary vitamin K consumption<br>Vitamin K intake was discovered to be positively associated with BMD cross-sectionally in women, but not in men.	Vitamin K intake has been shown to be associated with lower risk of fracture in older adults.	High
