# Supplementary material for: Nutrition, Physical Activity, and Dietary Supplementation to Prevent Bone Mineral Density Loss: A Food Pyramid
Source: Nutrients. 2021 Dec 24;14(1):74. doi: 10.3390/nu14010074 (PMC8746518; doi:10.3390/nu14010074)
Supplement: Supplementary file 1 [file nutrients-14-00074-s001.zip › nutrients-1519822-supplementary/Table S9b. Vitamin K supplementation.pdf]

Author	Type of study	Study period	Supplementation	Subjects	End point	Results	Conclusion	Strenght of evidence
Rønn et al. (2016) [131]	RCT double-blinded	2016	A tablet containing 375 µg menaquinone-7 (MK-7), taken once a day for 12 months	148 postmenopausal women (age 67.9 ± 4.6) with osteopenia, supplemented with calcium and vitamin D.	Effect of vitamin MK-7 on on undercarboxylated osteocalcin (ucOC), and bone mass and quality	ucOC decreased in the MK-7 group (−65.6%) (median (CI)) compared with the placebo group (−6.4%) after 3 months (P < 0.01).<br>Changes in aBMD after 12 months were small and did not differ between groups	Vitamin MK-7 preserves trabecular bone structure at the tibia.	High
Inaba et al. (2015) [130]	RCT double blinded	2015	Study 1: Four dosage group -0, 50, 100 or 200 µg daily for 4-wk<br>Study 2: placebo or 100 µg MK-7 for 12 wk	Study 1: 60 postmenopausal women aged 50-59 y<br>Study 2: 120 subjects aged 20-69 y	Effective minimum daily menaquinone-7 dose for improving osteocalcin gamma-carboxylation.	Menaquinone-7 increased the carboxylated osteocalcin/undercarboxylated osteocalcin ratio dose dependently, and significant effects were observed in both the 100 and 200 µg groups compared with the 0 µg group.	Daily MK-7 intake >100 µg was suggested to improve osteocalcin carboxylation.	High
Morato-Martinez et al. (2020) [132]	RCT double blinded	24 weeks	The participants were randomly assigned to 1 of 2 treatment groups: the experimental group (EG=33) or control group (CG=32).<br>The dairy product enriched with nutrients contained 80 µg of Vitamin K	65 healthy menopausal women at risk of osteoporosis or untreated osteopenia completed the 24-week study.<br>Both groups had a similar bone masses, BMDs and T-scores at the start of the study.	Compare the effect of consuming a dairy preparation to reconstitute, similar to yogurt, enriched in calcium, vitamin D, vitamin K, vitamin C, zinc, magnesium, L-leucine and probiotic on bone metabolism markers for 24 weeks	The experimental group showed a significantly increased bone mass compared to the control group.<br>At the end of the intervention, the EG increased its bone mass significantly compared to the CG (-0.01 ± 0.03 vs. 0.01 ± 0.03 kg; $p < 0.05$).	The consumption of an experimental dairy product resulted in a significant improvement in bone mass content and managed to mitigate the loss of bone mineral density, unlike in the control group.	High
Mott et al. (2019) [133]	Meta-analysis	2019	36 studies:<br>• 9 studies: Vitamin K1	36 Randomized controlled trials assessing oral	An update to the systematic review about	The odds of any clinical fracture were lower for vitamin K compared to	For post-menopausal or osteoporotic patients, there is no evidence that	High

			(from 100 to 10.000 µg, mean 2155±3294 µg)<br>• 22 studies: MK-4 (45 mg)<br>• 5 studies: MK-7 (mean 239±121 µg)	vitamin K supplementation that assessed bone mineral density or fractures in adult populations	Vitamin K affecting bone mineral density and fracture incidence	controls (OR, 0.72, 95%CI 0.55 to 0.95).<br>There was no difference in vertebral fractures between the groups (OR 0.96, 95%CI 0.83 to 1.11).<br>In the bone mineral density meta-analysis, percentage change from baseline at the lumbar spine was higher at 1 year (MD 0.93, 95%, CI −0.02 to 1.89) and 2 years (MD 1.63%, 95%CI 0.10 to 3.16) for vitamin K compared to controls	vitamin K affects bone mineral density or vertebral fractures; it may reduce clinical fractures; however, the evidence is insufficient to confirm this.	
